# Epigenomic identification of vernalization *cis*-regulatory elements in winter wheat

**DOI:** 10.1186/s13059-024-03342-3

**Published:** 2024-07-30

**Authors:** Yanhong Liu, Pan Liu, Lifeng Gao, Yushan Li, Xueni Ren, Jizeng Jia, Lei Wang, Xu Zheng, Yiping Tong, Hongcui Pei, Zefu Lu

**Affiliations:** 1 State Key Laboratory of Crop Gene Resources and Breeding, Institute of Crop Sciences, Chinese Academy of Agricultural Sciences, Beijing, 100081 China; 2 State Key Laboratory of Plant Environmental Resilience, College of Biological Sciences, China Agricultural University, Beijing, 100193 China; 3 College of Agronomy, Henan Agricultural University, Zhengzhou, 450046 China; 4 State Key Laboratory of Plant Cell and Chromosome Engineering, Institute of Genetics and Developmental Biology, Innovation Academy for Seed Design, Chinese Academy of Sciences, Beijing, 100101 China; 5 Center for Agricultural Resources Research, Institute of Genetics and Developmental Biology, Chinese Academy of Sciences, Shijiazhuang, 050022 China

**Keywords:** Vernalization, Regulatory elements, Epigenome, Winter wheat

## Abstract

**Background:**

Winter wheat undergoes vernalization, a process activated by prolonged exposure to low temperatures. During this phase, flowering signals are generated and transported to the apical meristems, stimulating the transition to the inflorescence meristem while inhibiting tiller bud elongation. Although some vernalization genes have been identified, the key *cis*-regulatory elements and precise mechanisms governing this process in wheat remain largely unknown.

**Results:**

In this study, we construct extensive epigenomic and transcriptomic profiling across multiple tissues—leaf, axillary bud, and shoot apex—during the vernalization of winter wheat. Epigenetic modifications play a crucial role in eliciting tissue-specific responses and sub-genome-divergent expressions during vernalization. Notably, we observe that H3K27me3 primarily regulates vernalization-induced genes and has limited influence on vernalization-repressed genes. The integration of these datasets enables the identification of 10,600 putative vernalization-related regulatory elements including distal accessible chromatin regions (ACRs) situated 30Kb upstream of *VRN3*, contributing to the construction of a comprehensive regulatory network. Furthermore, we discover that TaSPL7/15, integral components of the aging-related flowering pathway, interact with the *VRN1* promoter and *VRN3* distal regulatory elements. These interactions finely regulate their expressions, consequently impacting the vernalization process and flowering.

**Conclusions:**

Our study offers critical insights into wheat vernalization’s epigenomic dynamics and identifies the putative regulatory elements crucial for developing wheat germplasm with varied vernalization characteristics. It also establishes a vernalization-related transcriptional network, and uncovers that TaSPL7/15 from the aging pathway participates in vernalization by directly binding to the *VRN1* promoter and *VRN3* distal regulatory elements.

**Supplementary Information:**

The online version contains supplementary material available at 10.1186/s13059-024-03342-3.

## Background

Plants distinguish seasons by sensing temperature and photoperiod signals to flower and bear seeds at the right time [1]. Vernalization denotes the process by which plants coordinate the vegetative-to-reproductive transition or accelerate flowering via cold treatment [2]. In the case of winter wheat, vernalization is completed after cold winter, where wheat acquired the ability to switch from vegetative to reproductive growth [3]. Cold signals trigger shoot apical meristem (SAM) transition to reproductive SAMs post-vernalization [4, 5]. *Vernalization 3* (*VRN3*)/*Flowering locus T* (*FT*) orchestrates this transition by integrating various signals into SAM [6–8]. *VRN1*, an activator expressed in leaves and SAM, responds to cold exposure in wheat [9]. Conversely, *VRN2,* codes a zinc finger protein, represses flowering and its disruption leads to unvernalized flowering [10–12]. Additionally, *Agamous-like33* (*TaAGL33*) and *TaAGL22*, analogous to *Flowering locus C* (*FLC*), are downregulated by vernalization [13, 14]. *Wheat Suppressor of Overexpression of Constans 1* (*WSOC1*) links vernalization and photoperiod in wheat, enhancing flowering in *Arabidopsis* [15]. However, the regulation of these genes and the mechanism behind cold-induced transition in wheat remain elusive.

Upon sensing vernalization, plants transmit diverse signals from leaves to the shoot apical meristem (SAM), facilitating the phase transition [5, 16]. Subsequent to vernalization, plants maintain a “memory” of this process, flowering when season is favorable [17]. Epigenetic alterations are pivotal in maintaining this vernalization "memory" [17–23]. In *Arabidopsis*, H3K27me3 around the flowering inhibitor *Flowering Locus C* (*FLC*) is essential for vernalization memory, maintaining *FLC* repression [24, 25]. H3K27 tri-methylation, and H3K27 di-methylation increase, and H3K36 tri-methylation decreases after an extended period of cold [25, 26]. In wheat and barley, elevated H3K27me3 levels surround *VRN1* before vernalization, but this modification diminishes while H3K4me3 is established during vernalization [27, 28]. Various enhancers associated with *FT* play a pivotal role in its accurate expression via epigenomic modifications in *Arabidopsis* [29]. Nonetheless, the full scope of vernalization responses governed by epigenetic alterations across multiple tissues, and the involvement of novel regulatory elements or genes in vernalization, remains undiscovered.

Plants also employ a phase transition resembling an aging pathway that measures the endogenous developmental state, ensuring flowering occurs in adulthood [30, 31]. Critical components of this process are miRNA156 and *Squamosa Promoter Binding Protein-Like* (*SPL*) genes, regulating flowering through an age-dependent pathway [30–33]. Inhibition of *SPLs* through constitutive expression of *miRNA156* has been shown to cause late flowering in *Arabidopsis* [31]. AtSPLs activate miRNA172 expression and other pivotal genes like *LFY*, *FUL*, *AP1*, *AGL42*, and *SOC1*, facilitating flowering [30, 34, 35]. The miRNA156-*SPL*-miRNA172 cascade is considered to function downstream of *FT*/*FD* in *Arabidopsis* [30, 36]. However, the detail relationship between aging pathway and vernalization pathway is still elusive, especially in wheat.

Here, we generated epigenomic landscapes of leaf, axillary bud and shoot apex during vernalization in wheat, unveiling distinct responses across multiple tissues. We observed that chromatin accessibilities and histone modifications were tightly associated with transcriptional changes and sub-genome divergences. Whereas, H3K27me3 were associated with vernalization induced genes while exhibited limited effects on vernalization repressed genes. Our data facilitated the identification of vernalization-responsive *cis*-regulatory elements (CREs), allowing the construction of regulatory networks across various tissues. Specifically, typical regulatory elements were identified in 30Kb upstream of *VRN3* and bound by TaSPL7/15, and TaSPL7/15 were also found to bind *VRN1* promoter. These interactions are crucial for up-regulating *VRN1* and *VRN3* during vernalization, with defects in TaSPL7/15 delaying the vernalization response under cold conditions. These findings provide rich resources for understanding epigenetic regulations during vernalization and uncovering the intricate regulatory networks in wheat.

## Results

### Tissue-specific chromatin accessibility and transcriptome responses during wheat vernalization

**Fig. 1:**
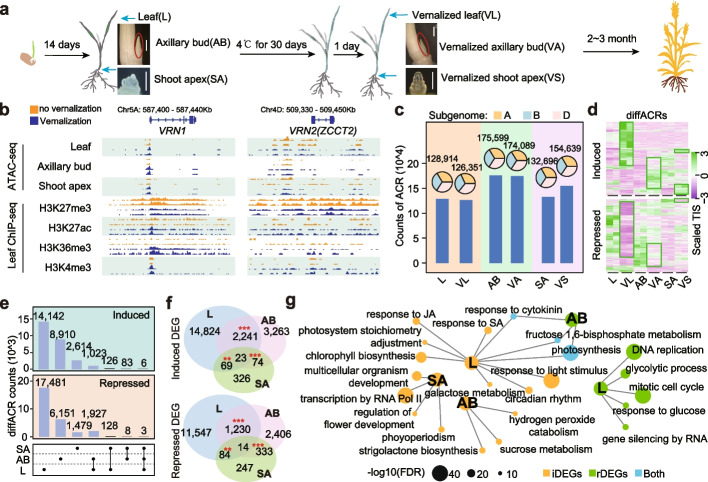
Tissue-specific chromatin accessibility and transcriptome responses during wheat vernalization. **a** Cultivation and sampling of plant materials. Wheat (*Triticum aestivum*; BBAADD, 2n = 6x = 42) cultivar AK58 was used in our study. Leaf (L), 0.2~0.3cm axillary bud (AB) and shoot apex (SA) were collected before (14 days after germination) and after vernalization (14 days after germination, 1 day in green house after 30 days after cold treatment and). The scale bar represents 1mm.** b** Chromatin accessibility and histone modifications of *VRN1* and *VRN2* during vernalization. **c** Counts and sub-genome distributions of Accessible Chromatin Regions (ACRs) in all 6 samples. **d** Chromatin accessibilities of differential ACRs in the 6 samples. Induced ACRs indicates ACRs with increased accessibility after vernalization and repressed ACRs indicates those with decreased accessibility**. e** Counts of overlapped differential ACRs from different combinations. **f** Induced and repressed Differentially Expressed Genes (DEGs) during vernalization. **g** Biological pathways enriched for different DEG groups

Vernalization triggers a systemic transition from vegetative to reproductive development, yet the responses of multiple tissues during this process remains unclear. We gathered samples of leaf, axillary bud, and shoot apex before and after vernalization to construct a comprehensive epigenomic atlas of different tissues. This included chromatin accessibility, transcriptome data from all 6 samples, and 4 key histone modifications (H3K27me3, H3K4me3, H3K36me3, and H3K27ac) in leaf and vernalized leaf (Fig. 1a and Additional file 1: Table S1) [37]. Generally, all transcriptomic and epigenomic data showed high reproducibility between biological replicates (Additional file 2: Fig. S1a-e). Active chromatin histone modifications (H3K27ac, H3K36me3 and H3K4me3) were positively correlated with each other, while being negatively associated with the repressive histone modification H3K27me3 (Additional file 2: Fig. S1f). Stronger chromatin accessibility signals were found in *VRN1* promoter, and histone modifications H3K27ac, H3K4me3, and H3K36me3 were also increased on A and B sub-genome, H3K27ac and H3K4me3 were increased on D sub-genome, while H3K27me3 was decreased after vernalization in all three sub-genomes (Fig. 1b and Additional file 2: Fig. S2a). All epigenomic modification changes of *VRN1* were consistent with its induced expression after vernalization (Additional file 2: Fig. S2b and 2c). In contrast, for the vernalization repressive gene *VRN2*, promoter chromatin accessibility in leaf and shoot apex were decreased, but not changed in axillary bud on A sub-genome *ZCCT1* and D sub-genome *ZCCT2,* consistent with the low expression level of *VRN2* after vernalization (Fig. 1b, Additional file 2: Fig. S2a and Additional file 2: Fig. S2d).

To clarify the differences of chromatin accessibility responses, we identified the accessible chromatin regions (ACRs) in each tissue. In total, 126K – 176K high-quality ACRs were identified in different tissues (Fig. 1c and Additional file 3: Table S2). Those ACRs were equally distributed among A, B, and D sub-genomes, and mostly located in the intergenic regions with an average ratio larger than 50%, followed by promoters (from 2Kb upstream to TSS), terminators (2Kb downstream of TTS), introns and exons (Fig. 1c and Additional file2: Fig. S3a). Differential ACRs responsive to vernalization were then identified among those ACRs, divided into induced ACRs and repressed ACRs, respectively. 15,297 induced ACRs and 19,539 repressed ACRs were identified in leaf, 10,022 induced ACRs and 8,089 repressed ACRs in axillary bud, and 2,829 induced ACRs and 1,618 repressed ACRs in shoot apex (Fig. 1d, e and Additional file 4: Table S3). Combining ACRs of leaf, axillary bud, shoot apex and root [38], we found 70%~84% of ACRs are common (Additional file 2: Fig. S3b). Further, we examined the vernalization responses of multiple tissues. There were more induced ACRs in leaf than the other two tissues with only 6.35% (1,023) were co-induced in axillary bud and 0.78% (126) in shoot apex (Fig. 1e). Similarly, there were more repressed ACRs in leaf with 9.74% (1,927) co-repressed in axillary bud and 0.65% (128) in shoot apex (Fig. 1e). Between 10% to 30% of these differential ACRs exist in only one tissue (Additional file 2: Fig. S3c), suggesting a tissue-specific nature of chromatin accessibility responses during wheat vernalization.

The considerable diversity of vernalization responses among multiple tissues was additionally affirmed by the transcriptomic data, which showed approximately 13.06% (2,241) co-induced Differentially Expressed Genes (DEGs) and 9.55% (1,230) co-repressed DEGs between leaf and axillary bud, while very few were co-regulated DEGs between shoot apex and other tissues (Fig. 1f and Additional file 5: Table S4). Considering the inherent differences in gene expression patterns among different tissues, we examined the expression patterns of genes (TPM > 1) across various tissues (Additional file 2: Fig. S4a). Only a few genes that were highly expressed in leaf before vernalization became highly expressed in axillary bud after vernalization, as well as in other tissues (Additional file 2: Fig. S4a). Additionally, the proportion of DEGs and differential ACR-related DEGs between different tissues is low, ranging from 0.02 to 0.21 (Additional file 2: Fig. S4b and Additional file 6: Table S5). This suggests that although tissue-specific expressions contribute to the differential responses to vernalization, the vernalization response is more likely tissue-specific.

DEGs in leaf were enriched in photosynthesis, light and hormones (Fig. 1g). In axillary bud, DEGs were enriched in “strigolactone biosynthetic”, “sucrose metabolic”, “hydrogen peroxide catabolic process” and “fructose 1,6-bisphosphate metabolic” (Fig. 1g), which might suggest a possibility that wheat regulates tillering by altering those pathways during cold treatment. In shoot apex, DEGs were involved in “flower development” and “organism development”, reflecting the transition from vegetative to reproductive phases (Fig. 1g). In summary, we established chromatin accessibility landscapes of multiple tissues during wheat vernalization and found that vernalization responses of leaf, axillary bud and shoot apex were dramatically different.

### Coordinate regulation of epigenomes and transcriptomes in response to vernalization

**Fig. 2:**
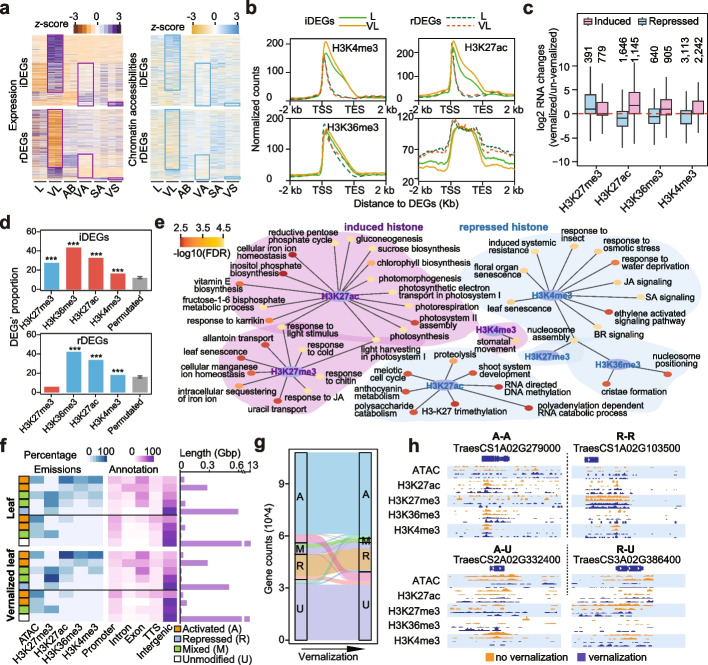
Alteration of histone modifications was critical for the expression changes during wheat vernalization. **a** Expression level and chromatin accessibility changes around DEGs. Expression levels were indicated by TPM. The normalized Tn5 transposome integration sites (TISs) from 500bp upstream to 50bp downstream of TSS were used to indicate chromatin accessibilities. iDEGs means induced DEGs, and rDEGs means repressed DEGs during cold vernalization. **b** Histone modification levels from 2Kb upstream to 2Kb downstream of DEGs. For H3K4me3, DEGs bearing H3K4me3 were first identified, and the same for H3K27ac, H3K36me3 and H3K27me3, respectively. **c** Expression level changes of genes with changed modification levels during vernalization. Induced represents genes with an increased level of modifications after vernalization, while repressed represents genes with a decreased modification level. **d** Proportions of DEGs among genes with changed modification levels. Red bars represent increased histone modifications after vernalization, blue bars represent decreased. “***” indicate *p* < 0.001. **e** Biological pathways enriched for genes with different modification changes. **f** Whole-genome chromatin states identified by ChromHMM in leaf and vernalized leaf. **g** Alternation of chromatin states during vernalization. **h** Represented samples of genes with changes chromatin states. “activated to unmodified” represents genes with activated chromatin state in leaf and unmodified chromatin state in vernalized leaf, similarly for “activated” to “activated”, “repressed” to “repressed” and “repressed” to “unmodified”

By comparing RNA-seq data before and after vernalization, we identified 17,157 vernalization-induced DEGs in leaf, 5,601 in axillary bud, and 492 in shoot apex, and 12,875 repressed DEGs in leaf, 3,983 in axillary bud, and 678 for shoot apex (Fig. 1f and Additional file 5: Table S4). Interestingly, the vernalization response of leaf was greater than that of axillary bud and shoot apex, which was reflected by the counts of differential ACRs and DEGs. It may attribute to their greater cellular diversity and relatively heightened exposure to environmental stimuli compared to axillary bud and apical meristems, which are enveloped within multiple layers of leaf. The chromatin accessibility around DEGs was generally correlated with their expression changes, and the chromatin accessibility of vernalization-induced DEGs’ promoters increased in leaf and axillary bud, while that of repressed DEGs in leaf, axillary bud and shoot apex decreased (Fig. 2a and Additional file 2: Fig. S5a). Compared with the random control, DEGs during vernalization has a strong correlation with the differential ACRs. 24%~26% of differential ACRs in leaf is related to DEGs, 23%~24% in axillary bud, and 5%~7% in shoot apex (Additional file 7: Table S6). The levels of active histone modifications increased after vernalization for the induced DEGs, and levels of H3K27ac and H3K36me3 decreased for the repressed DEGs (Fig. 2b and Additional file 2: Fig. S5b-c). H3K27me3 were decreased in the upstream and downstream regions of iDEGs, while only slightly increased in genebodies of rDEGs during cold vernalization (Fig. 2b). These findings suggest that the upregulated DEGs were linked with increased activated histone marks and a decrease in H3K27me3, while downregulated genes were mainly associated with the reduction of active histone modifications during vernalization.

To evaluate the effects of different histone modifications, we identified genes with changed histone modification levels during vernalization (Additional file 8: Table S7 and Additional file 9: Table S8). Genes with increased active modification(s) were usually vernalization-induced, while those with an increased repressive modification were reduced (Fig. 2c). More than 40% of genes with induced H3K27ac modifications were vernalization induced DEGs, followed by 32.7% with induced H3K36me3 and 16.3% with induced H3K4me3, while similar percentages were also found among genes with reduced modifications of those marks, respectively. However, although a high percentage of genes (27.3%) with reduced H3K27me3 modifications were induced DEGs, only a few repressed DEGs (5.8%) were found among genes with increased H3K27me3 (Fig. 2d). GO enrichment analysis revealed that disparate histone modifications are responsible for different biological pathways during vernalization. Genes with altered H3K27me3 were likely to regulate light and temperature signals and hormones. H3K27ac-related genes were involved in meristem development and cell cycle, H3K36me3-related genes in nucleosome assembly and positioning, and H3K4me3-related genes in floral organ development and hormones (Fig. 2e).

To further understand the transition of chromatin states during vernalization, we classified chromatin into 9 states using ChromHMM, a chromatin state annotation software based on multivariate hidden Markov model (HMM) [39, 40, 41, 42] (Fig. 2f). Each of the 9 chromatin states has varying degrees of chromatin accessibility and histone modifications (Fig. 2f). Essentially, we observed that regions with high levels of H3K4me3 and H3K36me3 were enriched in genic regions and those without these marks were found in promoter and intergenic regions (Fig. 2f). This observation is consistent with the understanding that H3K4me3 is a typical promoter histone modification [43, 44]. We then labeled regions with higher chromatin accessibility and active histone modifications as “activated”, and those with H3K27me3 as “repressed”, those with both kind of marks as “mixed” and regions that were not accessible and did not carry any of the four types of histone modifications as “unmodified” (Fig. 2f). Approximately 500-600MB regions were labeled as repressed chromatin state and 400MB regions were labeled as activated chromatin state (Fig. 2f). By combining the chromatin states of leaf and vernalized leaf, we delineated chromatin changes during vernalization and found most genes maintained their chromatin states after vernalization (85.9%). Among those that shifted, the majority converted from activated to unmodified, mixed to activated, mixed to repressed, repressed to unmodified, unmodified to activated, and unmodified to repressed, while only a few converted from activated to repressed, or repressed to activated (Fig. 2g, h). We hypothesized that genes in a mixed state, potentially arising from diverse cellular states in the bulk assay or bivalent modifications [45–47], were prone to manifest heightened sensitivity in response to cold vernalization treatment. Therefore, these results suggest that epigenetic modifications regulate wheat vernalization by affecting gene expression, with different histone modifications playing diverse roles.

### Sub-genome specific responses during wheat vernalization

**Fig. 3:**
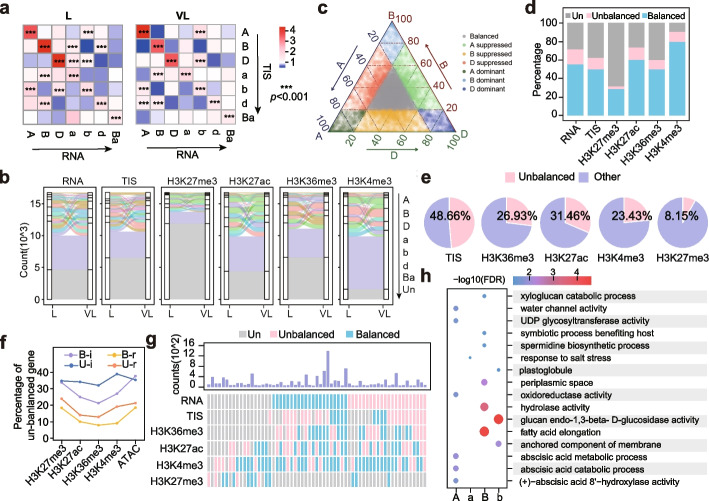
Sub-genome specific responses during wheat vernalization. **a** Sub-genome expression differentiation is associated with chromatin accessibility. Expression levels were indicated by TPM. The normalized Tn5 transposome integration sites (TISs) from 500bp upstream to 50bp downstream of TSS were used to indicate chromatin accessibilities. A, A dominant; B, B dominant; D, D dominant; a, a suppressed; b, b suppressed; d, d suppressed; Ba, balanced. *** indicated *p* < 0.001. **b** Changes in sub-genome differentiation states of expression, chromatin accessibility and histone modifications (H3K27me3, H3K27ac, H3K36me3 and H3K4me3) during vernalization. Un, represents not expressed (TPM <1), not accessible, or no such 4 modifications. **c** Sub-genome differentiation of expression responses during vernalization. **d** Percentage of genes with sub-genome differentiated responses in expression, chromatin accessibility and histone modifications during cold vernalization. “Balanced” represents balanced responses during vernalization. “Unbalanced” represents unbalanced responses during vernalization.** “**Un” represents not expressed (TPM < 1), not accessible, or no such 4 modifications. **e** Ratio of unbalanced modification responses in triads with unbalanced expression responses. **f** Proportion of genes with unbalanced expression responses in different groups. “B” represents balanced responses during vernalization. “U” represents unbalanced responses during vernalization. “i” indicates increased chromatin accessibility or histone modification, while “r” indicates decreased chromatin accessibility or histone modification. **g** Contributions of chromatin accessibility and histone modifications to sub-genome-differentiated expression responses. “Un” represents not expressed (TPM < 1) or no modification. Combinations with a number greater than or equal to 50 are shown in the figure and used for statistics. **h** Biological pathways enriched among sub-genome-specific responses genes

Common wheat is an allohexaploid composed of three sub-genomes (A, B and D), and the divergence in expression of homoeologous genes is critical for its enhanced environmental adaptivity [48]. The differences in expression were highly related to their promoter accessibility, indicating that promoter variations have a strong correlation with sub-genome divergences (Fig. 3a). We then classified the triad genes into A dominant (A), B dominant (B), D dominant (D), A suppressed (a), B suppressed (b), D suppressed (d) and balanced (Ba) based on their gene expression changes and promoter accessibility changes, respectively (Fig. 3b). Among the expressed genes (TPM >= 1 in at least one tissue), 55% showed balanced responses among the three sub-genomes, while 16% showed unbalanced responses (Fig. 3c, d and Additional file 2: Fig. S5d). We further profiled chromatin accessibility and histone modifications of homoeologous genes during vernalization and found that most of the triplets maintained the balanced mode, with around 3%-12% converting between balanced and unbalanced (A, B, D, a, b, d) patterns (Fig. 3c, d).

To further evaluate the correlation of epigenetic modifications to sub-genome divergent expressions during vernalization, we conducted statistical analysis on histone modification changes in triads with expression changes. Among triads with unbalanced expression changes, 48.66% were associated with unbalanced changes in chromatin accessibility, 31.46% with H3K27ac, 26.93% with H3K36me3 and 23.43% with H3K4me3, while only 8.15% with H3K27me3 (Fig. 3e and Additional file 10: Table S11). Triads with unbalanced chromatin accessibility and histone modification changes were more likely to exhibit unbalanced expression changes compared to balanced ones (Fig. 3f). By combining expression, chromatin accessibility, and the four types of histone modification changes during vernalization, we identified the correlations of chromatin accessibility and histone modifications to expression changes (Fig. 3g). By calculating the overlap between various epigenetic modification responses and gene expression responses, we discovered that chromatin accessibility, H3K27ac, H3K36me3, and H3K4me3 were more closely linked with unbalanced expression changes (Fig. 3g and Additional file 2: Fig. S5d). Furthermore, genes with unbalanced expression changes were enriched in different biological processes. For example, A-specific responsive genes were enriched in UDP glycosyltransferase activity, ABA catabolic and metabolic process, while B-specific responsive genes were enriched in fatty acid elongations (Fig. 3h). These results suggest that chromatin accessibility and histone modifications have a strong relationship with the unbalanced expression changes of sub-genomes during vernalization.

### Identification of putative vernalization-related regulatory elements

**Fig. 4:**
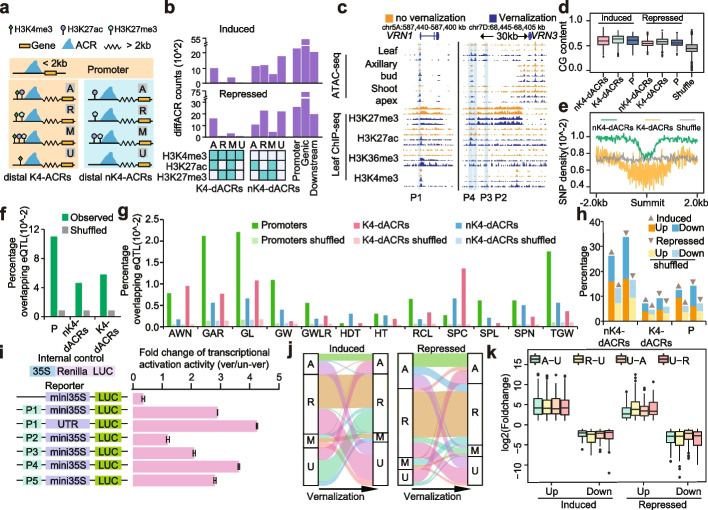
Identification of vernalization-related ACRs. **a** Strategies for identifying *cis*-regulatory elements (distal ACRs and promoters). A represents activated (H3K4me3 and H3K27ac); R represents repressed (H3K27me3); M represents mixed (both A and R); U represents unmodified. **b** Counts of vernalization-related K4-dACRs and nK4-dACRs identified based on chromatin accessibilities and histone modifications. **c** Genome coverage images showing the epigenomic modifications around *VRN1* and the 30Kb upstream distal ACRs of *VRN3*. P1~P4 are the fragments selected for the following experiments. **d-e** GC contents and SNP densities of identified regulatory elements. nK4-dACRs represents distal ACRs without H3K4me3; K4-dACRs represents distal ACRs neighboring to H3K4me3; P represents promoter. **f-g** Percentages of identified regulatory elements overlapping with eQTL. Shuffle represents the average percentage of randomly selected genomic regions (1000 times). SPC represents spike compactness; RCL represents red coleoptile; AWN represents awnedness; GWLR represents grain width to length ratio; SPN represents spikelet number per spike; SPL represents spike length; HT represents height; HDT represents heading date; GW represents grain weight; GAR represents grain area; GL represents grain length; TGW represents thousand grain weight. **h** Counts of DEGs with changed chromatin accessibilities during vernalization. Induced represents increased chromatin accessibility after vernalization; Repressed represents decreased chromatin accessibility after vernalization; Up represents up regulated gene; Down represents down regulated gene. nK4-dACRs represents distal ACRs without H3K4me3; K4-dACRs represents distal ACRs neighboring to H3K4me3. **i** Transcriptional regulatory activities of vernalization responsive distal ACRs through luciferase (LUC) activity assays in wheat protoplasts. The results depicted in the Fig 4i. represent the ratio of the regulatory activity of the fragment in the vernalized state compared to its activity in the non-vernalized state. Wheat protoplasts were extracted from leaf with/without vernalization (4℃ for 4 weeks). P1~P4 are the ACRs marked in Fig. 4c, and P5 is the ACR marked in Fig. S6a. **j** Chromatin state shift of nK4-dACRs during cold vernalization. **k** Expression changes of genes with chromatin state shift. A-U represents genes whose chromatin states are A before vernalization and U after vernalization, similarly for R-U, U-A and U-R

Distal *cis*-regulatory elements are critical for fine-tuning gene expression [49]. Through integrative genome-wide epigenetic profiling, we developed a strategy to identify putative regulatory elements (Fig. 4a). Firstly, we labeled ACRs located within 2Kb away from genes as promoters. H3K4me3 is usually considered to be the mark of promoter or unannotated promoters in the intergenic regions, while some studies also believe that H3K4me3 co-localizes with enhancer [50–52], which suggest that H3K4me3 is important to distinguish the types of ACRs. Therefore, we divided the distal ACRs based on the presence or absence of H3K4me3. ACRs beyond 2Kb from genes and surrounded by H3K4me3 were labeled as distal H3K4me3 associated ACRs (K4-ACRs), and ACRs beyond 2Kb from genes and not surrounded by H3K4me3 as distal no H3K4me3 associated ACRs (nK4-ACRs) (Fig. 4a). And we further pay more attentions to the nK4-ACRs. All distal ACRs were classified into 4 groups based on their nearby histone modifications: “activated” if they were next to the acetylation mark H3K27ac, “repressed” if they were next to H3K27me3, “mixed” if they were next to both kind of marks and “unmodified” if they lacked the 4 types of modifications (Fig. 4a). Active clusters were typically associated with high expression, while repressive ones were associated with low expression (Additional file 2: Fig. S5e). To identify putative vernalization-related regulatory elements, we focused on differential ACRs and classified them using the above strategy (Fig. 4b). Differential ACRs close to genes (within 2Kb of genes) were identified as promoters. Distal differential ACRs (dACRs, 2Kb away from genes), which are putative distal regulatory elements responsive to cold vernalizations, were also divided into distal differential K4-ACRs (K4-dACRs) and nK4-ACRs (nK4-dACRs) based on whether they were surrounded by H3K4me3 (Fig. 4b). A total of 3561 and 10,600 vernalization responsive K4-dACRs and nK4-dACRs were identified, respectively (Fig. 4b, Additional file 11: Table S12 and Additional file 12: Table S13). Taking *VRN-D3* as an example, the accessibilities of these regions increased after cold treatment and H3K27ac modifications were enriched around the corresponding ACRs, while H3K27me3 modifications decreased (Fig. 4c). We also observed that the H3K27me3 modifications around the differential ACRs extend to the *VRN3* genebody (Fig. 4c).

All these dACRs, along with the promoters, had higher GC contents compared to random intergenic regions (Fig. 4d). Additionally, the SNP density of these dACRs was lower than neighboring regions (Fig. 4e), suggesting their functional importance. Furthermore, we examined the GWAS signals [53] of heading date traits within a 1Kb range of the identified regulatory elements and found a significantly higher overlapping percentage compared to random genomic sequences. Moreover, many other important productivity trait-related GWAS signals were also enriched, including spike morphologies and grain traits (Fig. 4f, g). Consistently, genes associated with these vernalization responsive dACRs and promoters were more likely to exhibit differential expression compared to the random ones (Fig. 4h), confirming the regulatory activities of these regions.

We subsequently chose several dACRs, such as the *VRN3*-related ones (Fig. 4c), *WSOC1*-related ones (Additional file 2: Fig. S6a) and also *VRN1* promoter (Fig. 4c), to validate their potential transcriptional regulatory functions using luciferase (LUC) activity assays conducted in wheat protoplasts. Interestingly, in wheat protoplasts isolated from leaf subjected to cold vernalization, the LUC activities of these regulatory elements were enhanced (Fig. 4i). Comparable results were observed in cold treated tobacco leaf (Additional file 2: Fig. S6b). While when using accessible chromatin regions who were not responsive to vernalization, the relative activities did not show significant changes (Additional file 2: Fig. S6c-d).

In addition, we characterized the states of vernalization-responsive nK4-dACRs before and after vernalization. Among all these ACRs with state changes, the most common ones are transitioned from activated to unmodified, repressed to unmodified, unmodified to activated and unmodified to repressed, then mixed to repressed, and mixed to unmodified (Fig. 4j), suggesting a consistent pattern with that of genes (Fig. 2g). We then examined the expression changes of genes with modification transition during vernalization and found that gene expression changes were most strongly associated with chromatin accessibility, followed by histone modifications (Fig. 4k). These results suggest that the alteration of chromatin accessibility and histone modifications of regulatory elements, especially those CREs, were associated with genes’ expression.

### Divergence of vernalization responsive distal ACRs among sub-genomes

**Fig. 5:**
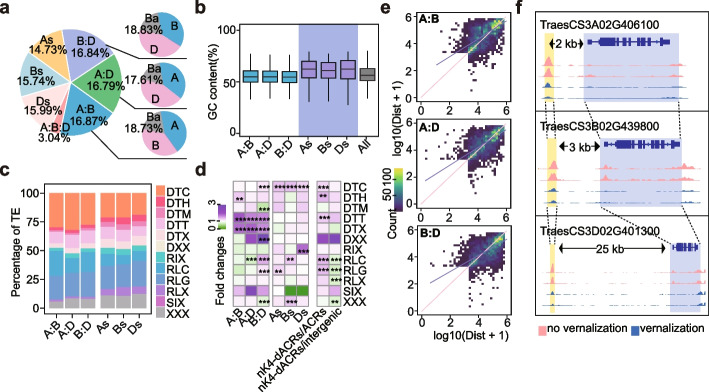
Sub-genome divergences of vernalization related distal ACRs. **a** Classification of vernalization related nK4-dACRs based on collinearity and homoeologous genes. **b** GC content of different type nK4-dACRs. A:B represents syntenic nK4-dACRs between A and B sub-genome, similarly for A:D and B:D. As represents A sub-genome specific nK4-dACRs, similarly for Bs and Ds. **c** TE enrichment of syntenic and sub-genome specific nK4-dACRs compared to TE enrichment in all nK4-dACRs. nK4-dACRs/ACRs represents the ratio of TE enrichment of nK4-dACRs to ACRs. nK4-dACRs/intergenic represents the ratio of TE enrichment of nK4-dACRs to intergenic regions. **d** Percentage of TE in syntenic and sub-genome-specific nK4-dACRs. **e** Distances between the syntenic nK4-dACRs and their corresponding genes. A:B represents syntenic nK4-dACRs between A and B sub-genomes, the abscissa indicated A sub-genome specific response and the ordinate indicated B sub-genome specific response, similarly for A:D and B:D. **f** Represented nK4-dACRs with different distances away from genes. “***” indicated *p* < 0.001

To further elucidate the divergences of vernalization responsive distal ACRs among sub-genomes, we identified homologous nK4-dACRs through pairwise comparisons (A:B, A:D and B:D). Approximately 53% of vernalization responsive nK4-dACRs were homologous and corresponded to the homoeologous genes (Fig. 5a). Among these homologous nK4-dACRs, around 82% exhibited diverse chromatin accessibilities (Fig. 5a). These findings indicate that nK4-dACRs exhibit greater sequence variations in comparison to neighboring genes. A total of 516, 624 and 600 sub-genome-specific nK4-dACRs were found in A, B and D sub-genomes, respectively. These sub-genome specific nK4-dACRs had a higher GC content than the common ones (Fig. 5b). To determine the origin of these sub-genome specific nK4-dACRs, we focused on the transposable elements (TEs) enriched by these specific nK4-dACRs. Common vernalization responsive nK4-dACRs had a higher overlap with DTT and DTX (14%-16%), while specific ones had a higher overlap with RLG, RLC and RIX (36-37%, Fig. 5c, d), respectively. These results suggest that retrotransposons played critical roles in the divergent evolution of vernalization responsive nK4-dACRs. Furthermore, we observed differences in the distances between syntenic nK4-dACRs and their corresponding genes among sub-genomes (Fig. 5e). Taking the one associated with *TraesCS3D02G401300* as an example, the nK4-dACR located 25 Kb away from the gene in D sub-genome was homologous to the promoter regions 2-3Kb upstream of the syntenic genes (Fig. 5f), indicating that some nK4-dACRs may originate from the promoter regions.

### TaSPL7/15 regulate vernalization by anchoring the promoter of *VRN1* and distal differential ACRs of *VRN3*

**Fig. 6:**
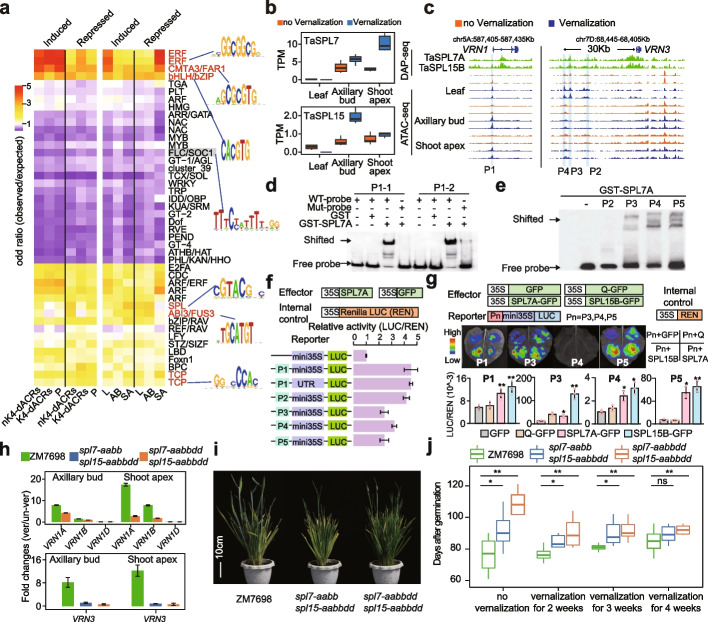
TaSPL7/15 regulate vernalization through binding the regulatory elements of *VRN1* and *VRN3*. **a** Key TFs enrichment of the putative vernalization-related regulatory elements. nK4-dACRs represents distal ACRs without H3K4me3; K4-dACRs represents distal ACRs neighboring to H3K4me3; P, promoter (2kb upstream of TSS). **b** The expression of *TaSPL7/15* were increased after vernalization in axillary bud and shoot apex. **c** Genome coverage image showing that TaSPL7 and TaSPL15 bind to the promoter of *VRN1* and distal ACRs of *VRN3*. P1~P4 are the fragments selected for the following experiments. **d-e** EMSA showing the binding of TaSPL7A to *VRN1* promoter (**d**) and *VRN3* distal ACRs (**e**). P1-1 and P1-2 are the two SPL binding sites within the *VRN1* promoter (Fig. S8d). **f-g** Validation of TaSPL7/15 binding and activation activity in wheat protoplasts (**f**) and tobacco leaf (**g**) using dual luciferase Reporter assay. Here, we selected some fragments for verification. P1~P4 are the ACRs marked in Fig. 6c, and P5 is the ACR marked in Fig. S8c. Statistical analyses are shown in the bottom panel and “*” indicates *p* < 0.05, “**” indicates *p* < 0.01 in Student’s *t*-tests. The relative activity was calculated by comparing the LUC/REN value of SPL to the LUC/REN value of GFP. **h** Induced expression of *VRN1* and *VRN3* were repressed in *spl7-aabbdd spl15-aabbdd* hexa-mutants. Fold changes represent the ratio of *VRN1* and *VRN3* expression ((7 days after vernalization) / (no vernalization)). Samples for RT-qPCR were obtained from plants grown 2 weeks (day for 16h at 22°C, night for 8h at 19°C) after germination and 7 days in greenhouse (day for 16h at 22°C, night for 8h at 19°C) after vernalization (plants grown 2 weeks in green house and vernalization for 3 weeks). All samples were taken between 9AM and 12AM (China Standard Time). **i** The late flowering phenotype of *spl7-aabbdd spl15-aabbdd* hexa-mutants (84 days after germination). **j** Statistics of heading time. Statistical analyses are shown in the bottom panel and “*” indicates *p* < 0.05, “**” indicates *p* < 0.01 in Student’s *t*-tests

To identify the key TFs binding to the vernalization-related regulatory elements, we constructed a regulatory network of 2,668,346 interactions, including 1,383 responsive TFs and 3,588 other responsive genes in the three tissues, based on the distribution of TF binding motifs in promoters and dACRs (Additional file 2: Fig. S7). We further examined the enrichment of 40 motif clusters from JASPR to determine TF binding preferences in promoters and distal ACRs [54]. TF binding motifs were generally consistent between induced and repressed differential ACRs (Fig. 6a), indicating both kinds of ACRs were under the control of similar TFs or TF families. TF binding motifs related to light signal response, including FAR1, FHY3, and PIF (Fig. 6a), were significantly enriched in all induced and repressed differential ACRs, suggesting that vernalization also recruits light signaling pathways [55], although only cold treatment was carried out in our experiments. TFs associated with plant hormone signaling and development-related TFs, including ARF, TCP, ERF, BPC, and SPL, showed higher motif enrichments, while MYB, Dof, and WRKY binding motifs were depleted (Fig. 6a). Interestingly, we also found that FLC/SOC1 binding motifs were depleted in all three tissues (Fig. 6a), indicating that those motifs were not activated immediately after cold vernalization.

**Fig. 7:**
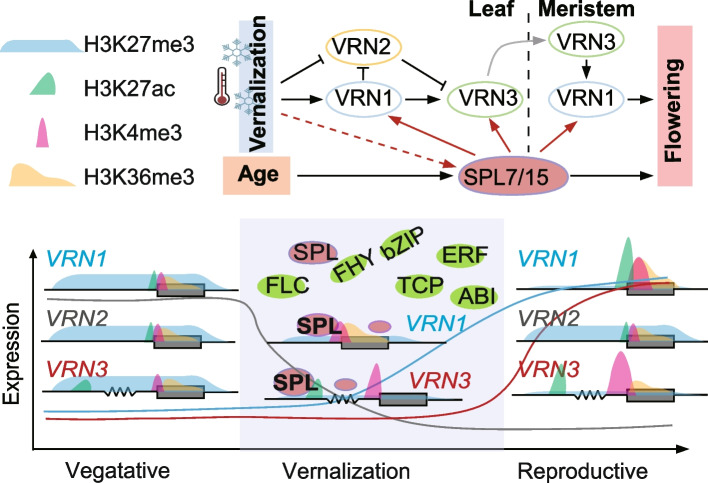
Schematic diagram showing the regulatory networks during wheat vernalization. During the vernalization process in wheat, the expression of *VRN1* and *VRN3* increases, while the expression of *VRN2* decreases. Concurrently, the modifications of H3K27ac, H3K36me3, and H3K4me3 on *VRN1* increase, whereas the level of H3K27me3 modification around *VRN1* decreases. The modifications of H3K36me3 and H3K4me3 on *VRN3* also increase, along with an increase in H3K27ac levels on the distal regulatory elements, while the surrounding H3K27me3 levels decrease. Numerous transcription factors are involved in the regulation of vernalization, among which SPL7 and SPL15 are induced by vernalization. These factors regulate wheat vernalization by directly binding to the *VRN1* promoter and *VRN3* distal regulatory elements, thereby activating their expression in leaf and apical meristem

SPL and ABI3 binding motifs were specifically enriched in the nK4-dACRs (Fig. 6a). SPLs are important plant-specific TFs that control wheat development and stress responses, but little is known about their involvement in vernalization. We observed that the expression of *TaSPL14/4* were repressed in leaf during wheat vernalization, and *TaSPL8/15/9* were repressed in axillary bud, while *TaSPL3/5/7/8/15/11* were induced in axillary bud and shoot apex, suggesting that this family may play diverse roles during wheat vernalization (Fig. 6b, Additional file 2: Fig. S8a and Additional file 13: Table S14). Given that *TaSPL7/15* are the orthologous genes of Arabidopsis *SPL9* and rice *OsSPL14/IPA1*, which has been proved to be important for age dependent flowering regulation and plant architecture regulation [30, 56], we paid further attention to *TaSPL7/15*. Also, it’s reported that they were related to wheat tillering and panicle development [38]. Using our published DAP-seq results [38, 57] and DAP-qPCR (Additional file 2: Fig. S8b), we found TaSPL7A and TaSPL15B binding regions were enriched in the differential ACRs (Additional file 2: Fig. S8c). Moreover, clear binding peaks were observed in regulatory regions of *VRN1*, *VRN3* and *WSOC1* (Fig. 6c and Additional file 2: Fig. S8d). To further confirm these interactions, we conducted EMSA, Dual-Luciferase Reporter assay and DAP-qPCR, which demonstrated that TaSPL7/15 could bind specifically to the promoter of *VRN1*, and the distal ACRs of *VRN3* and *WSOC1,* and activated the expressions of related genes (Fig. 6d-g and Additional file 2: Fig. S8e-h). Consistently, the vernalization responses of *VRN1* and *VRN3* were dramatically inhibited in axillary bud and shoot apex in *spl7-aabbdd spl15-aabbdd* hexa-mutants (Fig. 6h). It is reported that the mutation of SPL binding sites in *VRN1* promoter affect its expression [58]. These results collectively suggested that TaSPL7/15 participates in wheat vernalization by regulating the expression of *VRN1* and *VRN3*. Yet, upon evaluating the vernalization responses of *spl7-aabbdd spl15-aabbdd* hexa-mutants, our findings revealed a 30-day delay in flowering compared to the wildtype without vernalization, with a 10-day delay observed under 2 and 3 weeks of vernalization, while extending the cold treatments to 4 weeks didn't yield noticeable differences (Fig. 6i, j and Additional file 2: Fig. S9a). Also, it’s found that leaf of *spl7-aabbdd spl15-aabbdd* hexa-mutants increased (Additional file 2: Fig. S9b-c), which is similar with the more rosette leaf and late flowering phenotype of spl9 mutants in *Arabidopsis* [30]. We then examined the expression changes of *VRN1* and *VRN3* in un-vernalized wheat, revealing their up-regulation before flowering (Additional file2: Fig. S10a). This up-regulation of *VRN1* and *VRN3* still relied on TaSPL7/15 (Additional file 2: Fig. S10b). And the vernalization response of *VRN1* in the *spl7-aabbdd spl15-aabbdd* hexa-mutants were significantly repressed compared to wild type (Additional file 2: Fig. S10c). Additionally, *VRN3* hardly responded during the vernalization process and only began to response after vernalization was completed (Additional file 2: Fig. S10c). These findings suggest that TaSPL7/15 is essential for the up-regulation of *VRN1* and *VRN3*. Consequently, the accumulation of *VRN1* due to vernalization effectively mitigated the delayed flowering observed in *spl7-aabbdd spl15-aabbdd* hexa-mutants. This highlights the significance of TaSPL7/15 induced by vernalization (Fig. 6h) in promoting flowering by modulating the expression of *VRN1* and *VRN3* across multiple tissues, integrating signals from vernalization and other pathways (Fig. 7).

## Discussion

Vernalization is the process where plants experience a period of low temperatures [2], and has similarities and differences with cold stress which was defined as a major environmental factor that seriously affects plant growth and development [59]. During vernalization, winter wheat undergoes vegetative-to-reproductive transition and accelerates flowering [7, 10, 60]. In addition to the developmental transition of meristems, the development of leaf and tillers is also inhibited during cold vernalizations. Previous studies have primarily focused on the roles of several key *VRN* genes [6, 7, 15, 60–62]. We found that the responses of leaf, axillary bud and shoot apex were dramatically different. For examples, sucrose-related pathways were enriched in leaf and axillary bud, which is consistent with the notion that sucrose accumulation can enhance wheat’s resistances to cold. All these results suggest that the responses of different tissues during cold vernalization are diverse.

Epigenetic modifications are considered to play critical roles in maintaining vernalization memory [25, 26]. In wheat and barley, H3K27me3 levels around *VRN1* decreased, while H3K4me3 levels increased during cold vernalization [27, 28]. However, for the vernalization repressor *VRN2*, H3K27me3 levels did not increase despite its expression being repressed. We also observed that different types of histone modifications may have disparate roles during vernalization (Fig. 2e). The decrease of H3K27me3 has a greater impact on the induced expression of genes compared to the increase of H3K27me3 on repressed expression during wheat vernalization. Additionally, the transitions between activating and repressive marks were less frequent than the changes between active or repressive histone modifications and unmodified groups, indicating that the establishment of new modifications typically occurs after the depletion of the previous modifications.

Enhancers play an important role in the spatiotemporal expression of genes and plant development [63]. In *Arabidopsis*, *FT*-associated enhancers have been reported to regulate flowering through epigenetic modifications [29]. By combining multiple omics data, we identified putative vernalization-related *cis*-regulatory elements (CREs) in multiple tissues in wheat and confirmed their sensitivity to vernalization. These CREs exhibited high diversity among different sub-genomes, contributing to the sub-genome divergent expressions observed in the allohexaploid wheat. Interestingly, although a CRE located 30Kb upstream of *VRN3* was identified, the sequence of this CRE is not homologous to those around *FT* in *Arabidopsis*, suggesting a different origin and evolution for this CRE compared to its counterpart in *Arabidopsis*.

*SPLs* are important transcriptional factors that control plant development in various stages and stress responses. The miRNA156-*SPL*-miRNA172 cascade is a well-known aging pathway that regulates flowering in *Arabidopsis*. However, it is still unclear whether *SPLs* are involved in vernalization. In wheat, the expressions of different *SPLs* were differentially regulated in multiple tissues during vernalization, and SPL binding motifs were enriched in the CREs identified in all three tissues. *TaSPL7A/15B*, the orthologs of rice *Ideal Plant Architecture 1* (*IPA1*) were reported to regulate plant height and tillering [38]. Consistently, we found that TaSPL7A/15B could directly bind to *VRN1* promoter through the binding sites and distal regulatory elements of *VRN3.* Defects in these genes resulted in delayed flowering, particularly when shortening the cold vernalization period. We verified that while TaSPL7/15 is induced by vernalization, it participates in the wheat vernalization process by regulating *VRN1* and *VRN3*. Although the deficiency of *SPL7* and *SPL15* delays the expression rise of *VRN1* and *VRN3*, the late flowering phenotype of *spl7a/b/d spl15a/b/d* hexa-mutants can be suppressed by extending the period of cold vernalization. This indicates that there is an SPL7/15-independent pathway that can activate *VRN1* and *VRN3* expression. Besides, *TaSPL7A/15B* is highly expressed in axillary bud and shoot apex but has low expressed in leaf, suggesting that there may be other *SPLs* involved in regulating vernalization signal perceptions in leaf. In summary, our study has constructed an epigenomic landscape of multiple tissues during vernalization, identified the regulatory elements, and revealed the novel roles of *SPLs* in vernalization. Our work not only sheds light on understanding the responsive regulatory networks during cold vernalization but also identifies crucial regulatory elements and transcription factors essential for improving wheat vernalization traits.

## Conclusions

Here, we constructed an epigenomic and transcriptomic map for multiple tissues including leaf, axillary bud, and shoot apex before and after vernalization to uncover the vernalization responses of multiple tissues in wheat. Both atlas of chromatin accessibility and gene expression suggesting a tissue-specific nature of chromatin accessibility responses during wheat vernalization (Fig. 1d-f). And the change of epigenome modifications during vernalization are responsible for gene expressions (Fig. 2a-c) and sub-genome divergent expression (Fig. 3). Interestingly, we found that genes whose expression was up-regulated during vernalization were related to increased activated histone marks (H3K27ac, H3K4me3 and H3K36me3) and a decrease in H3K27me3, while genes whose expression was down-regulated were mainly related to the reduction of active histone modifications (Fig. 2d). Combining maps of chromatin accessibility, histone modifications and gene expression, we also identified vernalization-related regulatory elements and related transcription factors. Further, we found that TaSPL7/15 participate in wheat vernalization process by directly anchoring the *VRN1* promoter and 30Kb upstream regulatory elements of *VRN3.* In summary, we uncovered the distributed vernalization responses through epigenomic and transcriptomic map of multiple tissues in winter wheat, unveiled the vernalization-related regulatory elements, and found that TaSPL7/15 from the aging pathway promote wheat flowering by regulating gene expression of *VRN1* and *VRN3.* Our research yields extensive data resources and valuable insights into unraveling the regulatory mechanisms governing wheat vernalization. Additionally, it pinpoints CREs suitable as targets for gene editing, paving the way for engineering wheat germplasm with diverse vernalization traits.

## Methods

### Plant material

Wheat (*Triticum aestivum*; BBAADD, 2n = 6x = 42) cultivar AK58 was used in our study. Materials without vernalization were obtained from plants grown for 2 weeks (day for 16h at 22°C, night for 8h at 19°C, light starts at 8 AM in the morning and darkness starts at 24 PM.) after germination. Materials with vernalization were obtained from plants that were planted in a greenhouse and subjected to 1 month of vernalization in a cold chamber (4℃, 16 h light/8 h dark), followed by growth in the greenhouse (day for 16h at 22°C, night for 8h at 19°C) for 1 day. Leaf were directly collected from the plants, while axillary bud and shoot apex were dissected under a microscope. All samples, including leaf, axillary bud and shoot apex, were taken between 9 AM and 12 AM (China Standard Time), that is 1-3 hours after starting the light (Our light cycle is from 8 AM to 24 PM, and the dark cycle is from 24 PM to 8 AM.).

ZM7698, and transgenic plants (*spl7-aabb spl15-aabbdd* and *spl7-aabbdd spl15-aabbdd*) with vernalization were grown 2 weeks (day for 16h at 22°C, night for 8h at 19°C) after germination and vernalization for 2, 3 or 4 weeks in cold chamber (4℃, 16 h light/8 h dark). Samples for RT-qPCR were obtained from plants grown 2 weeks (day for 16h at 22°C, night for 8h at 19°C) after germination and 1 day in greenhouse (day for 16h at 22°C, night for 8h at 19°C) after vernalization (3 weeks). All samples were taken between 9 AM and 12 AM (China Standard Time).

All samples before and after vernalization are basically guaranteed to only have vernalization treatment (low temperature treatment), excluding the influence of light and rhythm.

### RNA-seq

The collected materials were flash-frozen using liquid nitrogen. Total RNA was extracted using TRIzol^TM^ Reagent (Invitrogen, 15596-026). The RNA-Seq library was constructed by Berry Genomics (Beijing, China), and sequenced with Illumina NovaSeq 6000 platform. All sequencing data were 150bp paired-end reads.

### ATAC-seq

ATAC-seq was performed using our previously established protocol [64]. 1g frozen sample was minced in 1mL ice lysis buffer (15 mM Tris-HCl pH 7.5, 20 mM NaCl, 80 mM KCl, 0.5 mM spermine, 5 mM 2-Mercaptoethanol, 0.2% TritonX-100). The slurry containing the nuclei extract was filtered twice through a 40 μm filter. The crude nuclei containing DAPI (sigma, D9542) was loaded onto a flow cytometer (BD FACSCanto) for selection. The nuclei pellet was obtained by centrifugation and washed with Tris-Mg buffer (10 mM Tris-HCl pH 8.0, 5 mM MgCl_2_) and Tn5 transposomes in 40 μl TTBL buffer (Vazyme, TD501) were added for a 30-minute incubation at 37°C. Subsequently, the integration products were purified using the NEB Monarch™ DNA Cleanup Kit (T1030S) and library amplification was performed using NEB Next Ultra II Q5 master mix (M0544L). The amplified libraries were purified using Hieff NGS^®^ DNA Selection Beads (Yeasen, 12601ES03).

### ChIP-seq

ChIP-seq was performed as previously described [65] using antibodies against H3K27me3 (Abcam, ab6002), H3K4me3 (millipore, 07-473), H3K27ac (abclonal, A7253) and H3K36me3 (Abcam, ab9050). Libraries were constructed using the Kit (TransGen Biotech, KP201-02) and sequenced by Annoroad Gene Technology (Beijing, China). All libraries were sequenced on the Illumina NovaSeq 6000 platform to produce 150-bp paired-end reads.

### Processing of RNA-seq data

For data quality control, we used fastp v0.21.0 [66], and reads were aligned to the wheat reference genome (IWGSC RefSeq v1.0) [67] using hisat2 (https://daehwankimlab.github.io/hisat2/) with default parameters. BAM files were sorted using SAMtools v1.3.1 [68], read counts were quantified using FeatureCounts [69], and TPM (Transcripts Per Million) values were calculated using TPMCalculator [70]. Differential expressed genes (DEGs) were identified using DESeq2 [71] (*p*-value < 0.01 and Fold change > 2).

### Processing of ATAC-seq data

For data quality control, we used fastp v0.21.0 [66]. Reads were aligned to the wheat reference genome (IWGSC RefSeq v1.0) [67] using Bowtie v2.3.5 [72] (bowtie2 -X 1000 --very-sensitive). SAMtools v1.3.1 [68] was used to sort and filter bam (-q 10). Clonal duplicates were removed using Picard v2.16.0 (http://broadinstitute.github.io/picard/). Peaks were called using MACS2 v2.2.6 [73] (macs2 --keep-dup all --nomodel --extsizes 150 --shift -75). Raw peaks were divided into 150 bp bins with 50 bp overlapping, filtered based on Tn5 integration site density, and merged using bedtools with the “-d 200” parameter. Peaks overlapping with input and aligned to plant chloroplast DNA (NCBI) were discarded to ensure the identification of high-quality ACRs.

Differential ACRs were identified depending on the number of Tn5 transposome insertion sites (TISs) within each ACR. TISs were normalized by the total number of mapping reads as the library size. ACRs were divided into bins(200 bp), and DESeq2 [71] was used to find the difference interval based on TIS in each bin. Merge adjacent difference intervals and filter part of intervals using DESeq2 [71] to get high-quality differential ACRs.

### Processing of ChIP-seq data

We used fastp v0.21.0 [66] for data quality control and Bowtie v2.3.5 [72] to align reads to wheat reference genome (IWGSC RefSeq v1.0) [67] (bowtie2 -X 1000 --very-sensitive). SAMtools v1.3.1 [68] was used to sort and filter bam (-q 10), Picard v2.16.0 (http://broadinstitute.github.io/picard/) was used to remove clonal duplicates. Genes with changed modification levels were identified depending on the coverages of specific intervals. The interval from TSS to downstream 500bp for H3K27ac, H3K36me3 and H3K4me3, and genebody for H3K27me3. Read counts were normalized by the total number of mapping reads as the library size, and DESeq2 [71] was used to find genes with changed modification levels.

### Identification of chromatin status

ChromHMM, a chromatin state annotation software [39, 40], was used to do chromatin state annotation. Peaks of ATAC-seq, H3K4me3, H3K36me3, H3K27me3 and H3K27ac were used as input files to train the model, and then divide the chromatin states of each region on the whole genome. We identified 9 chromatin states with different chromatin accessibility degree and histone modifications, arranged vertically in 9 rows (Fig. 2f) [41, 42]. The Emissions are horizontal representation of chromatin accessibility and different histone modifications.

### Quantitative reverse transcription PCR (RT-qPCR)

All samples for RT-qPCR were frozen in liquid nitrogen, and total RNA was extracted using TRIzol^TM^ Reagent (Invitrogen, 15596-026). cDNA synthesis was carried out using the FastKing RT Kit (TIANGEN, KR116-02). Each RT-qPCR reaction utilized 10ng of cDNA and 1x TB Green Premix Ex Taq (TaKaRa, RR420). All quantitative materials are noted in the figure legends. All RT-qPCR primers were listed in Additional file 14: Table S13 [6, 7, 11].

### DNA affinity purification quantitative PCR (DAP-qPCR)

DAP-qPCR was performed as previously published with modification [38]. The HALO-SPL7A and HALO-SPL15B protein were generated using the TNT SP6 Coupled Reticulocyte Lysate System (Promega, L4600) according to the manufacturer’s protocol. The protein was then incubated with Magne-HALO Tag beads (Promega, G7282) for 1 h at 25 °C in 1× PBS with 0.005% Nonidet P-40 (PBST). After bound protein were washed with PBST and treated with DNaseI, the HALO-SPL7A and HALO-SPL15B protein were incubated with 500 μg of ultrasonicated genomic DNA library for 1 h at 25 °C. Then the beads were washed and recovered by V-ELUTE Gel Mini Purification Kit (Zoman, China, ZPV202). The ultrasonicated genomic DNA library was used as DNA input control for the qPCR reaction, three replicates of the fold enrichment on differential ACRs by SPL were calculated against input. Used primers in this experiment are listed in Additional file 14: Table S15.

### Electrophoretic mobility shift assay (EMSA)

The GST fused TaSPL7A protein were expressed in Escherichia coli BL21 (DE3) (Transgen, CD701-01), and then purified with BeaverBeadsTM GSH (Beaver, 70601-5) following manufacturer’s instructions [38]. DNA probes of 40-50 bp length were chemically synthesized and labeled with biotin at their 5’-end (Additional file 14: Table S15; BGI, China). The binding reactions and DNA gel-shift assays were performed according to the manufacturer’s instructions of LightShift Chemiluminescent EMSA Kit (Thermo Fisher Scientific, 20148). Briefly, 1 μg GST-TaSPL7A protein is incubated with DNA probe at room temperature for 20 minutes, the protein-DNA complexes are separated by electrophoresis using a native polyacrylamide gel. The gel was then transferred onto the Nylon Membrane. Membranes were imaged using a Biostep Celvin S Chemiluminescence Imager device after crosslink transferred DNA to membrane.

As for the GST fused TaSPL7A protein were expressed in Escherichia coli BL21 (DE3) (Transgen, CD701-01), and then purified with BeaverBeadsTM GSH (Beaver, 70601-5) following manufacturer’s instructions [38]. DNA probes (40-50 bp) containing the SPL binding motif GTAC and the GTAC mutation to AAAA were labeled and 5’biotin modification was synthesized in Beijing Genomics institution. Electrophoretic Mobility Shift Assays (EMSAs) were performed as LightShift® Chemiluminescent EMSA Kit (Thermo, 20148). Each experiment was independently replicated three times.

### Dual luciferase reporter assay

Dual luciferase reporter gene assay was performed in *N. benthamiana* leaf and wheat protoplasts. For the assay in *N. benthamiana* leaf, the regulatory elements of P1-P5 were amplified from AK58 genomic DNA and cloned into the pGreenII 0800-LUC vector as reporter plasmids. The CDS of *SPL7A* and *Q* were cloned in frame into the pEG1300-GFP vector as effector plasmids. *N. benthamiana* plants of 4-week-old were co-infiltrated with *A. tumefaciens* strain GV3101 harboring different combinations of these plasmids. The dual-luciferase reporter assay system (Yeasen, China) was used to quantify LUC and REN activities 48h after transfected according to the manufacturer’s instructions. For the assay in wheat protoplasts, the regulatory elements of P1-P5 were inserted into *mini35S::LUC* vector, the mixed plasmids containing 35S::*TaSPL7A*/GFP, *mini35S-Pn::LUC*, and pRTL in a ratio of 7:1:1 were introduced into wheat leaf protoplasts as described [74], while plasmids containing *35S::GFP*, pRTL, and *mini35S::LUC* as a negative control. The dual-luciferase reporter assay system (Yeasen, China) was used to quantify LUC and REN activities 16h after transfected and three independent replications were conducted.

## Peer review information

Wenjing She was the primary editor of this article and managed its editorial process and peer review in collaboration with the rest of the editorial team.

## Review history

The review history is available as Additional file 15.

### Supplementary Information

Additional file 1: Table S1. Statistical information of ATAC-seq, ChIP-seq and RNA-seq data.

Additional file 2: All supplementary figures included in this article. Figure S1. Repeatability between biological replicates and correlations between different histone modifications. Figure S2. Histone modifications and gene expressions of *VRN* genes. Figure S3. Characteristics of ACRs. Figure S4. The tissue specific expression patterns during vernalization. Figure S5. Epigenetic modifications and gene expression. Figure S6. Regulatory elements and verification of their activities. Figure S7. Response of transcription factor families during vernalization. Figure S8. TaSPL7/15 participated in wheat vernalization. Figure S9. Heading time and leaf counts of ZM7698 and *spl7-aabbdd spl15-aabbdd* hexa-mutant. Figure S10. Relative expression of *VRN1* and *VRN3* in leaf, axillary bud and shoot apex.

Additional file 3: Table S2. The identified ACRs from different tissues.

Additional file 4: Table S3. Vernalization-responsive differential ACRs.

Additional file 5: Table S4. Vernalization-responsive differentially expressed genes.

Additional file 6: Table S5. The proportion of vernalization-responsive genes and diffACR-related vernalization-responsive genes among genes with altered tissue expression patterns.

Additional file 7: Table S6. Different ACRs related to DEGs in various tissues.

Additional file 8: Table S7. Genes with changed histone modification levels during vernalization.

Additional file 9: Table S8. List of genes with different chromatin modifications.

Additional file 10: Table S9. Vernalization response of triads genes at the expression and epigenomic levels.

Additional file 11: Table S10. Vernalization related distal regulatory elements without H3K4me3.

Additional file 12: Table S11. Vernalization related distal regulatory elements with H3K4me3.

Additional file 13: Table S12. Gene IDs of *SPL* genes in this study.

Additional file 14: Table S13. Primers used for RT-qPCR, DAP-qPCR and vector construction.

Additional file 15. Review history.

## Data Availability

All sequenced data in this study are available from the Gene Expression Omnibus (GEO) database under the accession code GSE232430 [37]. SPL DAP-seq data are downloaded from Genome Sequence Archive under accession number PRJCA007017 [57]. Important scripts and genome-wide chromatin states information used in our study were publicly available on GitHub at https://github.com/WheatEpigenomics/WheatVernalization [42] under the MIT license and Zenodo (https://doi.org/10.5281/zenodo.12633435 [40]).
