# Supplementary material for: Epigenomic identification of vernalization cis-regulatory elements in winter wheat
Source: Genome Biol. 2024 Jul 30;25:200. doi: 10.1186/s13059-024-03342-3 (PMC11290141; doi:10.1186/s13059-024-03342-3)
Supplement: Supplementary file 2 — Additional file 2: All supplementary figures included in this article. Figure S1. Repeatability between biological replicates and correlations between different histone modifications. Figure S2. Histone modifications and gene expressions of VRN genes. Figure S3. Characteristics of ACRs. Figure S4. The tissue specific expression patterns during vernalization. Figure S5. Epigenetic modifications and gene expression. Figure S6. Regulatory elements and verification of their activities. Figure S7. Response of transcription factor families during vernalization. Figure S8. TaSPL7/15 participated in wheat vernalization. Figure S9. Heading time and leaf counts of ZM7698 and spl7-aabbdd spl15-aabbdd hexa-mutant. Figure S10. Relative expression of VRN1 and VRN3 in leaf, axillary bud and shoot apex. [file 13059_2024_3342_MOESM2_ESM.pdf]

Fig. S1

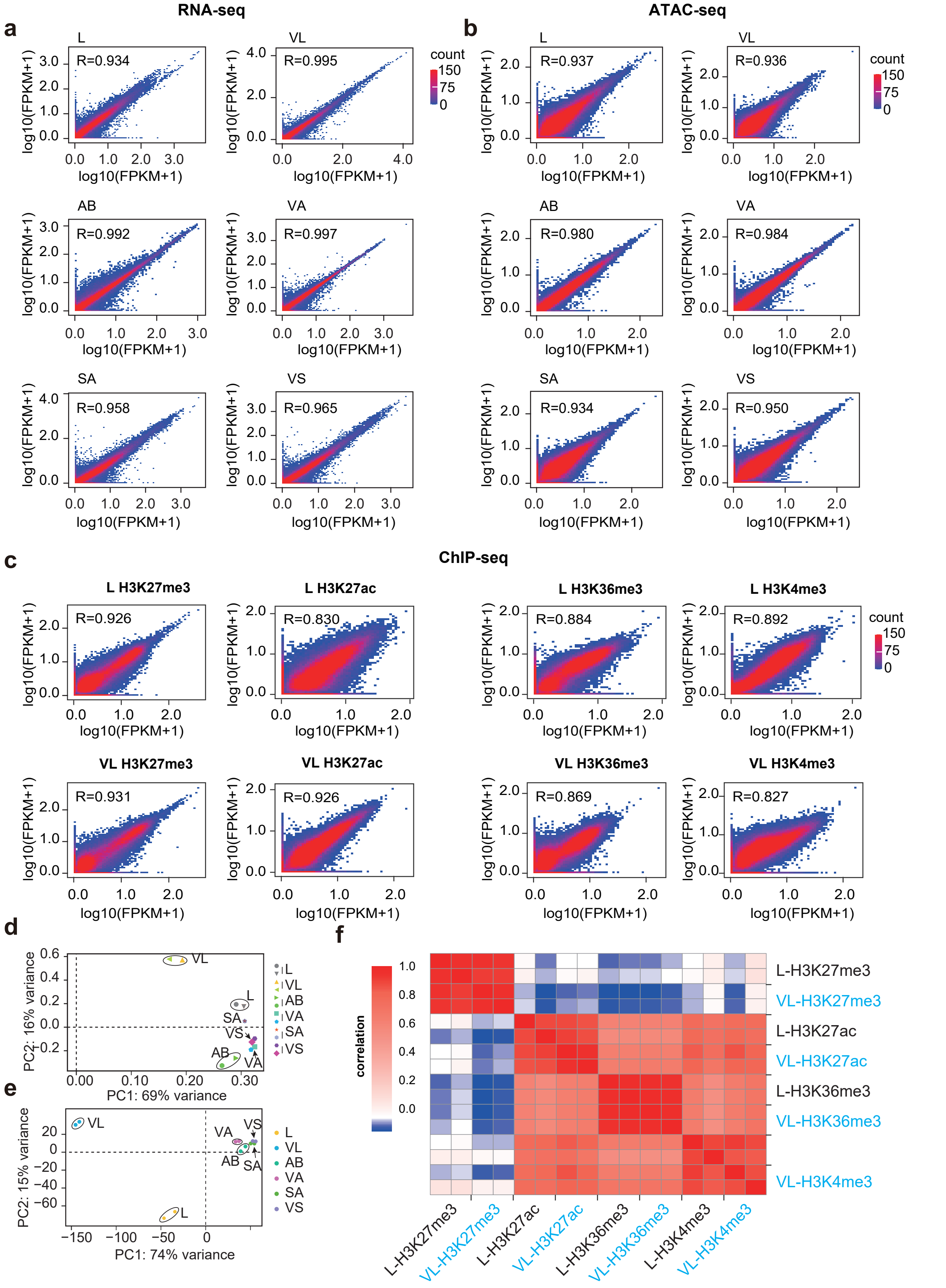

Fig. S1. Repeatability between biological replicates and correlations between different histone modifications.

a-c. Reproducibility between biological replicates. **d.** PCA of all samples (ATAC-seq based). **e.** PCA of all samples (RNA-seq based). L means leaf, VL means vernalized leaf, AB means axillary bud, VA means vernalized axillary bud, SA means shoot apex, and VS means vernalized shoot apex. **f.** Correlations between different histone modifications.

Fig. S2

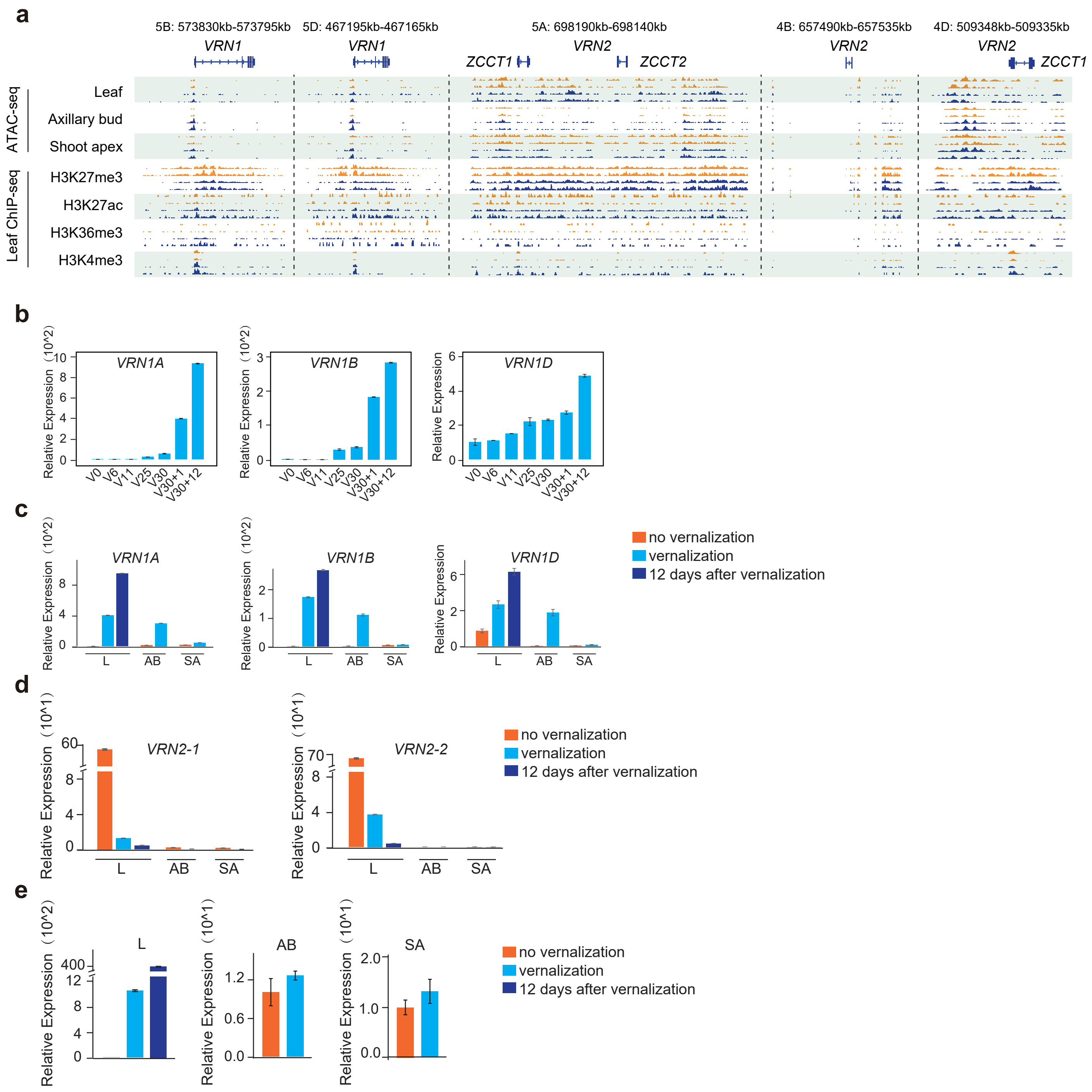

Fig. S2. Histone modifications and gene expressions of *VRN* genes.
a. Histone modifications of *VRN1* and *VRN2*. **b.** Expressions of *VRN1* in AK58 leaf at different stages. V0 means growing for 2 weeks in greenhouse after germination without vernalization, V30 means growing in greenhouse for 2 weeks after germination and vernalization for 30 days, and V30+1 means growing in green-house for 2 weeks after germination, then vernalization for 30 days and grown in green house for 1 day, and so on. **c-e.** Expressions of *VRN1* (**c**), *VRN2* (**d**), *VRN3* (**e**) genes in multiple tissues during vernalization. L means leaf, AB means axillary bud, and SA means shoot apex.

Fig. S3

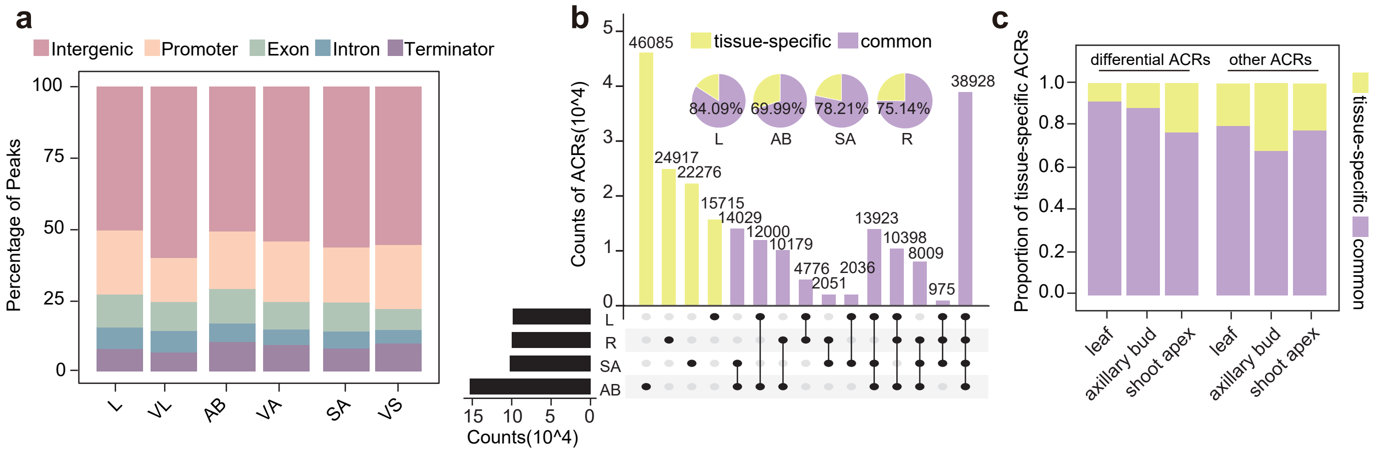

Fig. S3. Characteristics of ACRs.

a.The distribution of ACRs in different genomic regions. Promoter represent region from 2Kb upstream to 100bp downstream of Transcriptional Start Sites (TSSs). Terminator is regions from 100bp upstream to 2Kb downstream of Transcriptional End Sites (TESs). L means leaf, VL means vernalized leaf, AB means axillary bud, VA means vernalized axillary bud, SA means shoot apex, and VS means vernalized shoot apex. **b.** Distributions of ACRs identified across different tissues. **c.** Proportion of tissue-specific ACRs. ACRs subtracted the differential ACRs were labeled as other ACRs. "tissue-specific" represents ACRs that only exist in this organization. "common" represents ACRs that exist in at least two organizations. All analyzes were calculated based on ACRs of leaf, axillary bud and shoot apex.

Fig. S4

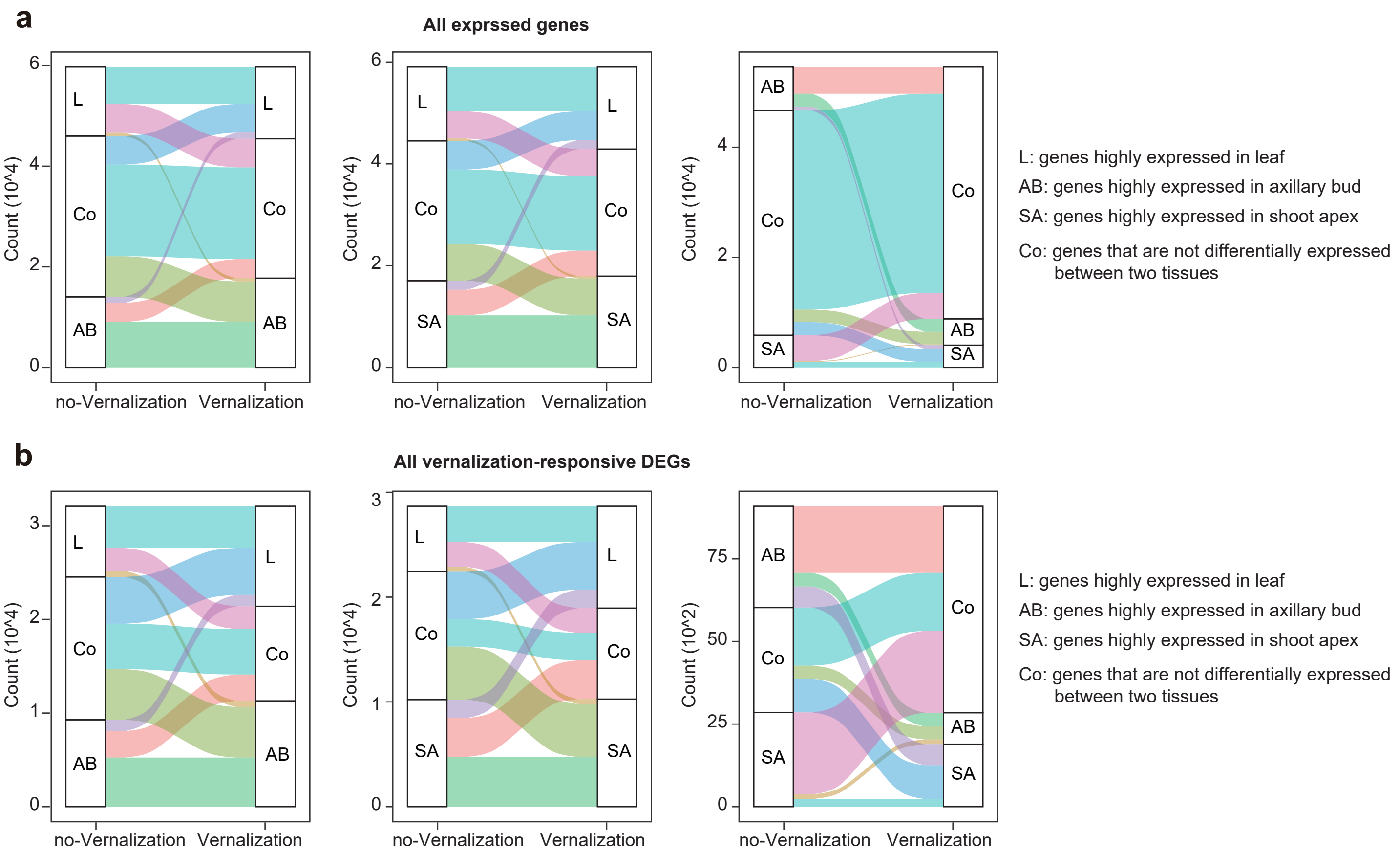

Fig. S4. The tissue specific expression patterns during vernalization.
a. The tissue specific expression patterns during vernalization of all expressed genes. Pairwise comparisons were performed for each two tissue pairs, and the conditions before and after vernalization have been identified. L means genes highly expressed in leaf. AB means genes highly expressed in axillary bud. SA means genes highly expressed in shoot apex. Co means genes that are not differentially expressed between the two tissues. Differentially expressed genes (DEGs) were identified using DESeq2 with Fold change > 2 and *p-value* < 0.01. All expressed genes indicated genes whose TPM > 1 in any tissue before or after vernalization. **b.** The gene expression pattern of vernalization-responsive DEGs. All these DEGs are part of the expressed genes in **a.**

Fig. S5

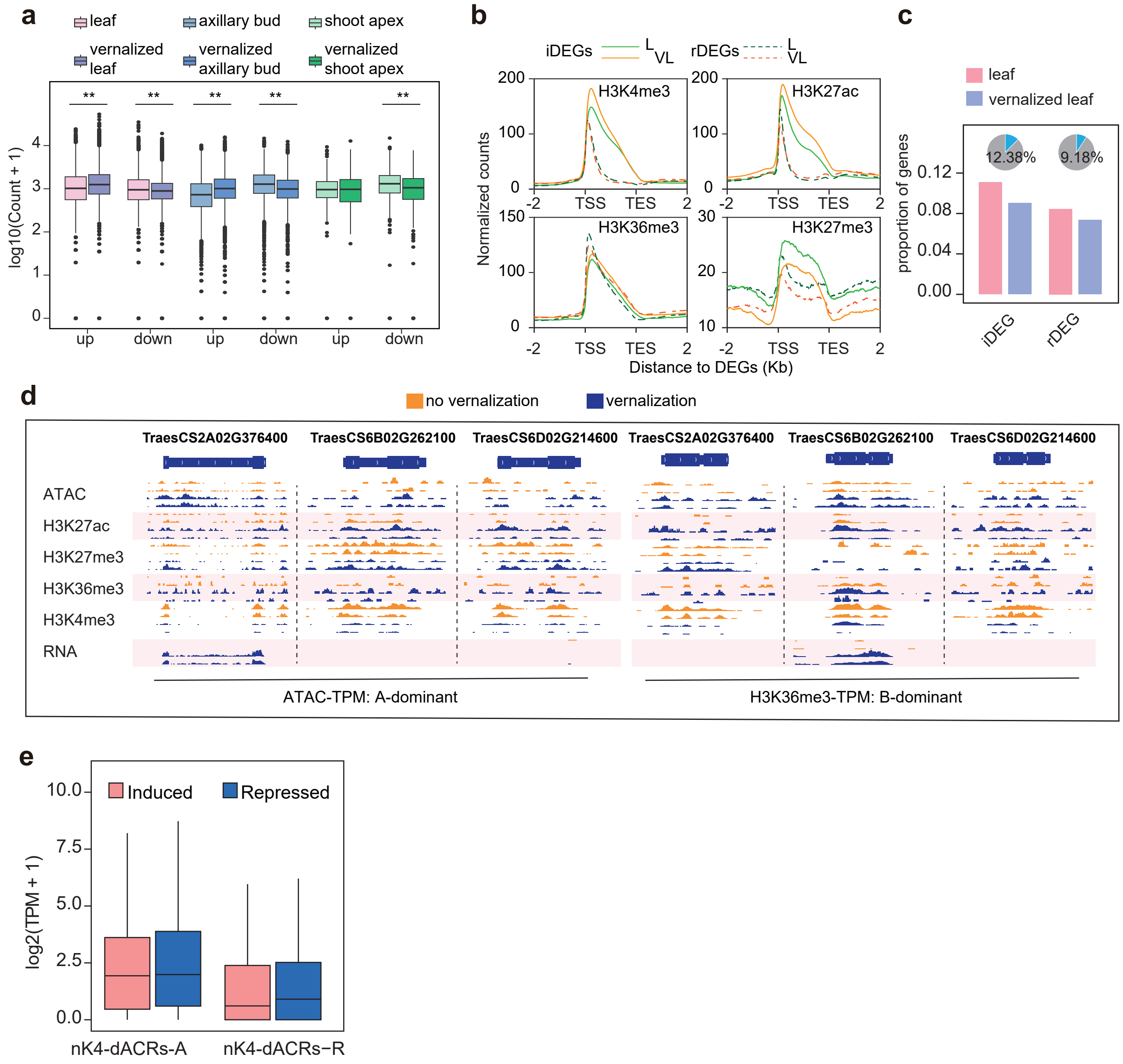

Fig. S5. Epigenetic modifications and gene expression.
a. Chromatin accessibility of differentially expressed genes in different tissues. Chromatin accessibility was characterized by the number of Tn5 insertion sites, which was normalized to the size of the ATAC-seq library. The ordinate count represents the number of insertion sites after normalization. up means up-regulated DEGs, and down means down-regulated DEGs. **b.** The histone modification levels of DEGs. iDEGs means all vernalization induced DEGs and rDEGs means vernalization repressed DEGs. **c.** The proportion of H3K27me3-modified genes among up-regulated and down-regulated genes. To determine whether the genes is modified by H3K27me3, the H3K27me3 ChIP-seq reads from 2kb upstream to 2kb downstream of genes were used to make a matrix and k-mean clustering were used to identify genes with higher read densities. iDEGs means up-regulated genes, and rDEGs means down-regulated genes during vernalization. **d.** Chromatin accessibility, histone modifications and gene expression of subgenome bias genes. A subgenome dominant response of ATAC-seq and TPM (left). B subgenome dominant response of H3K36me3 and TPM (right). **e.** Expression levels of distal nK4-ACRs associated genes with different chromatin states. nK4-dACRs, distal differential ACRs without H3K4me3. A and R represent the chromatin states defined in **Fig. 4b**. Induced, distal ACRs with increased chromatin accessibilities; Repressed, distal ACRs with repressed chromatin accessibilities.

Fig. S6

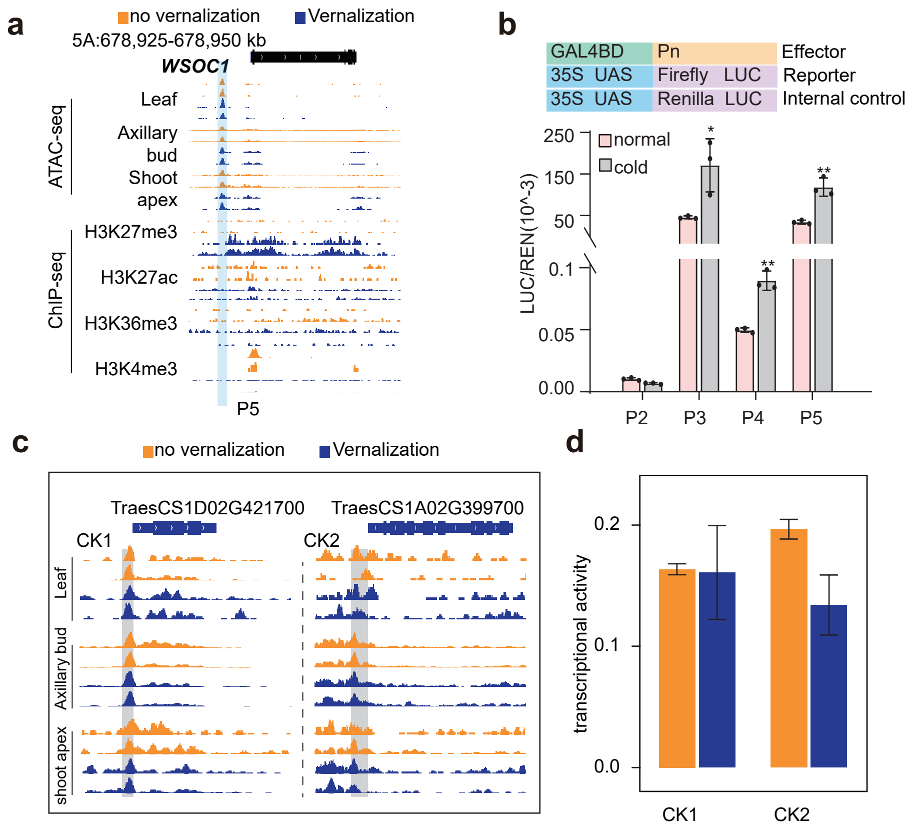

Fig. S6. **Regulatory elements and verification of their activities.**
a. Regulatory elements of *WSOC1*. **b.** Verification of transcriptional regulatory activities of these dACRs through luciferase (LUC) activity assays in tobacco leaves. **c.** IGV screenshots of two vernalization insensitive ACRs. CK1 and CK2 are accessible regions that do not respond to vernalization. **d.** Transcriptional regulatory activities of CK1 and CK2 through luciferase (LUC) activity assays in wheat protoplasts.

Fig. S7

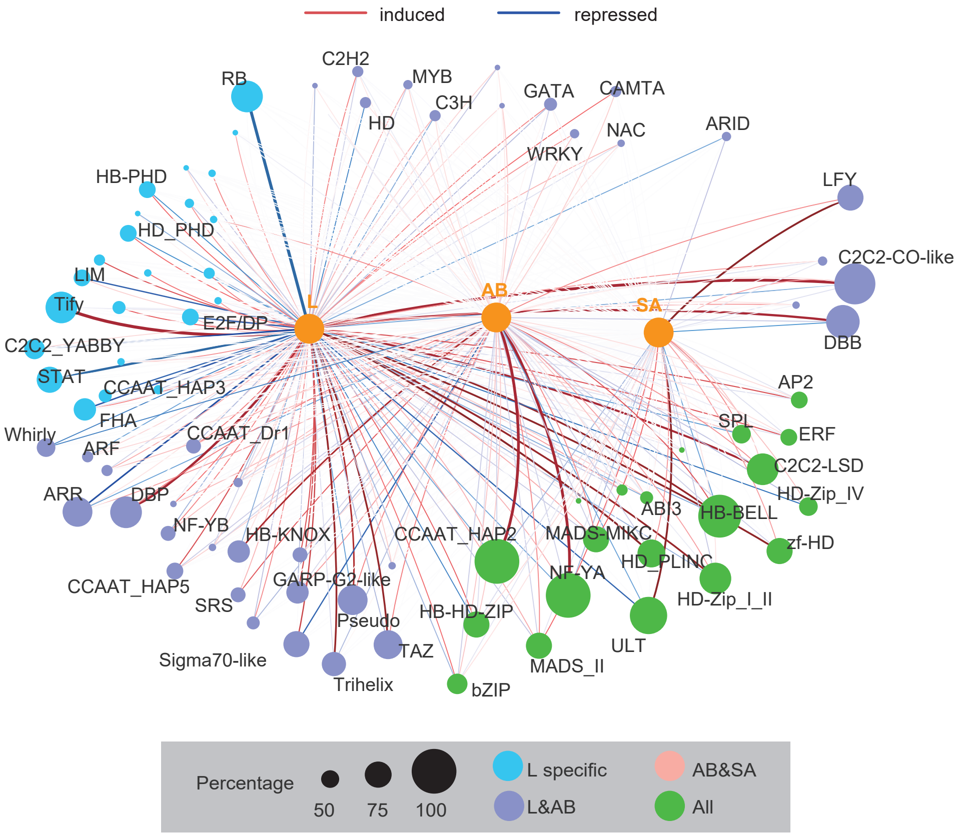

Fig. S7. Response of transcription factor families during vernalization.
Red lines indicate positively regulations while blue lines indicate negatively regulations. Darker colors and thicker connecting lines represent the number of interactions mediated by the transcription factor families. Percentage represents the ratio of the total number of differentially expressed genes to the entire TF family during vernalization. Points of different colors represent different categories, L specific, only responses in L, AB&SA, responses in AB and SA, L&AB, responses in L and SA, All, responses in L, AB and SA. L means leaf, AB means axillary bud, SA shoot apex.

Fig. S8

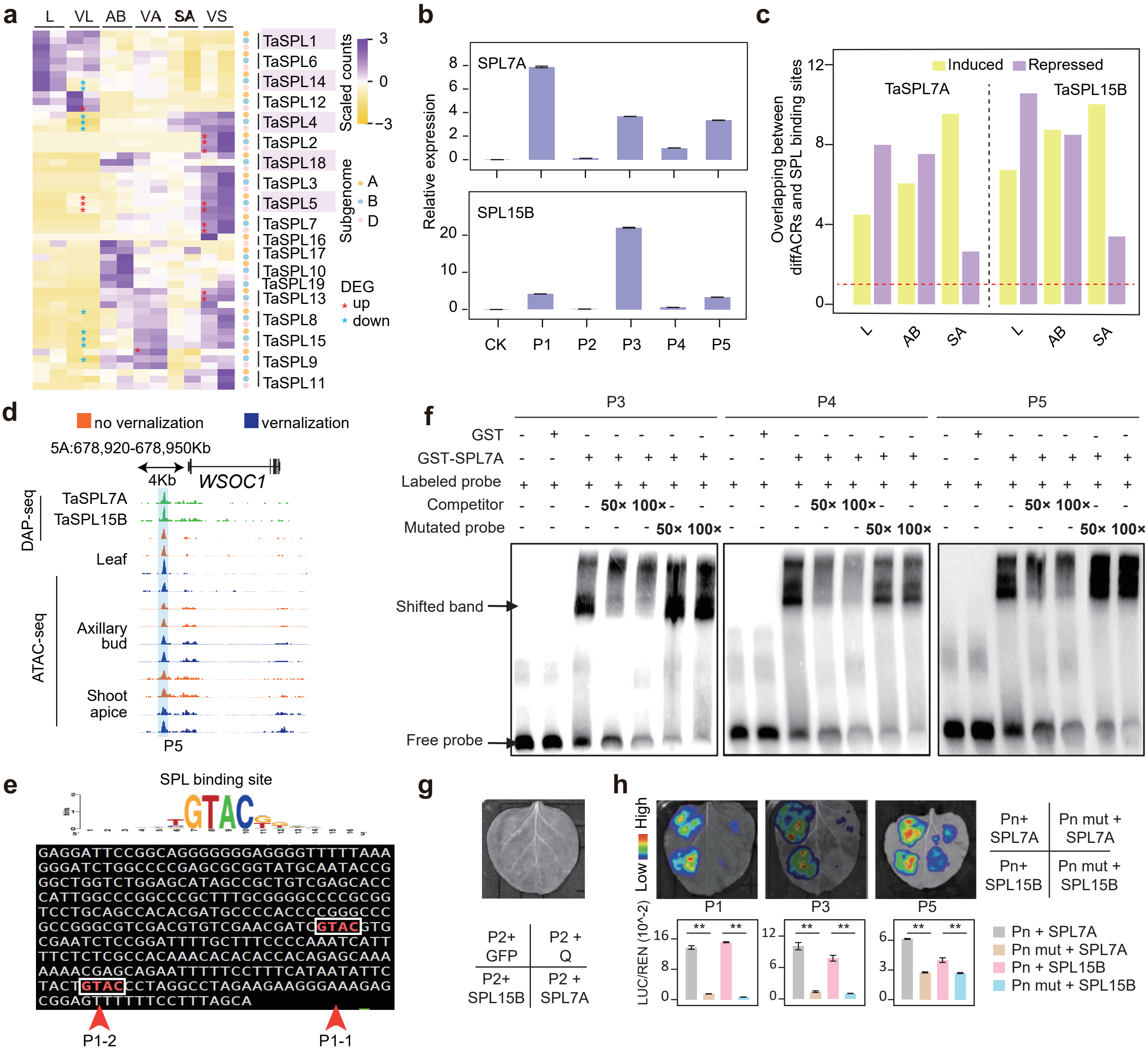

Fig. S8. *TaSPL7/15* participated in wheat vernalization.
a. TPM of SPL genes in different tissues. L, for leaf. VL, for vernalized leaf. AB, for axillary bud. VA, for vernalized axillary bud. SA, for shoot apex. VS, for vernalized shoot apex. **b.** Quantification of peaks using DAP-qPCR. P1-P4 were regions marked in **Fig. 6c**, and P5 was marked in **Fig. S8c**. **c.** Overlapping between TaSPL7 and TaSPL15 binding sites and differential ACRs. The red dot line indicated the rate of permutated random regions. **d.** A distal ACR 4kb upstream of *WSOC1*. **e.** Two different SPL binding sites within P1. **e.** EMSA of P3, P4 and P5. P3 and P4 stand for the fragments marked in **Fig. 6**. **f.** Validation of TaSPL7/15 specific binding. **g.** Validation of TaSPL7/15 specific binding. P2 is the ACR marked in **Fig. 6c**, and P5 is the ACR marked in **Fig. S6a**. Pn mut represents the Pn fragment with GTAC mutated. **h.** Validation of TaSPL7/15 specific binding. P1 and P3 are ACRs marked in **Fig. 6c**, and P5 is the ACR marked in **Fig. S6a**. Pn mut represents the Pn fragment with GTAC mutated.

Fig. S9

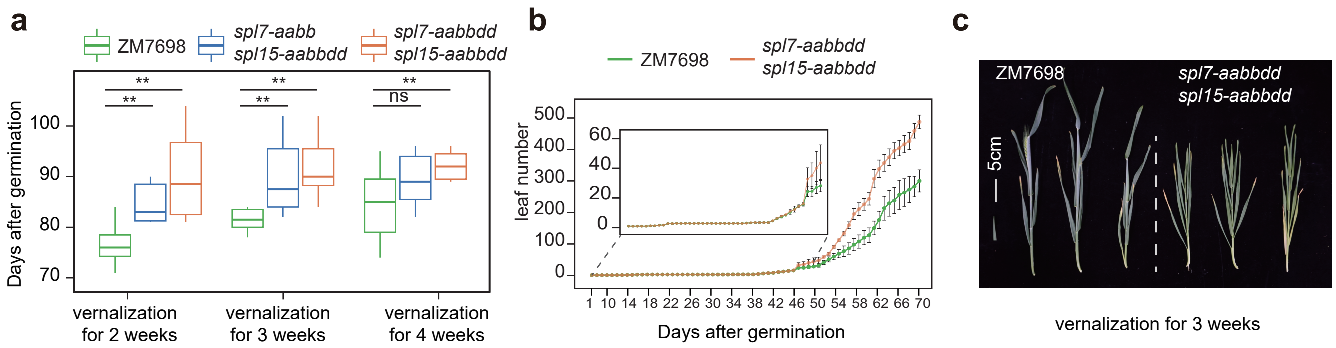

Fig. S9. Heading time and leaf counts of ZM7698 and *spl7-aabbdd spl15-aabbdd* hexa-mutant.
a. Heading times of ZM7698, *spl7-aabb spl15-aabbdd*, and *spl7-aabbdd spl15-aabbdd* mutants. All plants were treated with 2, 3, and 4 weeks of vernalization (4°C, 16 h light/8 h dark) after growing in the greenhouse (day for 16h at 22°C, night for 8h at 19°C) for 2 weeks. After vernalization, the plants continued to grow in the greenhouse (day for 16h at 22°C, night for 8h at 19°C), and the heading time of the earliest tiller of each plant was documented. **b.** Leaf counts of ZM7698 and *spl7-aabbdd spl15-aabbdd* hexa-mutant from germination to heading. All plants were treated with 3 weeks of vernalization (4°C, 16 h light/8 h dark) after growing in the greenhouse (day for 16h at 22°C, night for 8h at 19°C) for 2 weeks. After vernalization, all plants continued to grow in the greenhouse (day for 16h at 22°C, night for 8h at 19°C). The total leaf counts of all branches were recorded every two days until the heading of ZM7698. **c.** Phenotypes of single tillers. The plants were grown under the same conditions as described in b. The photos were taken when ZM7698 was about to head (70 days after germination).

Fig. S10

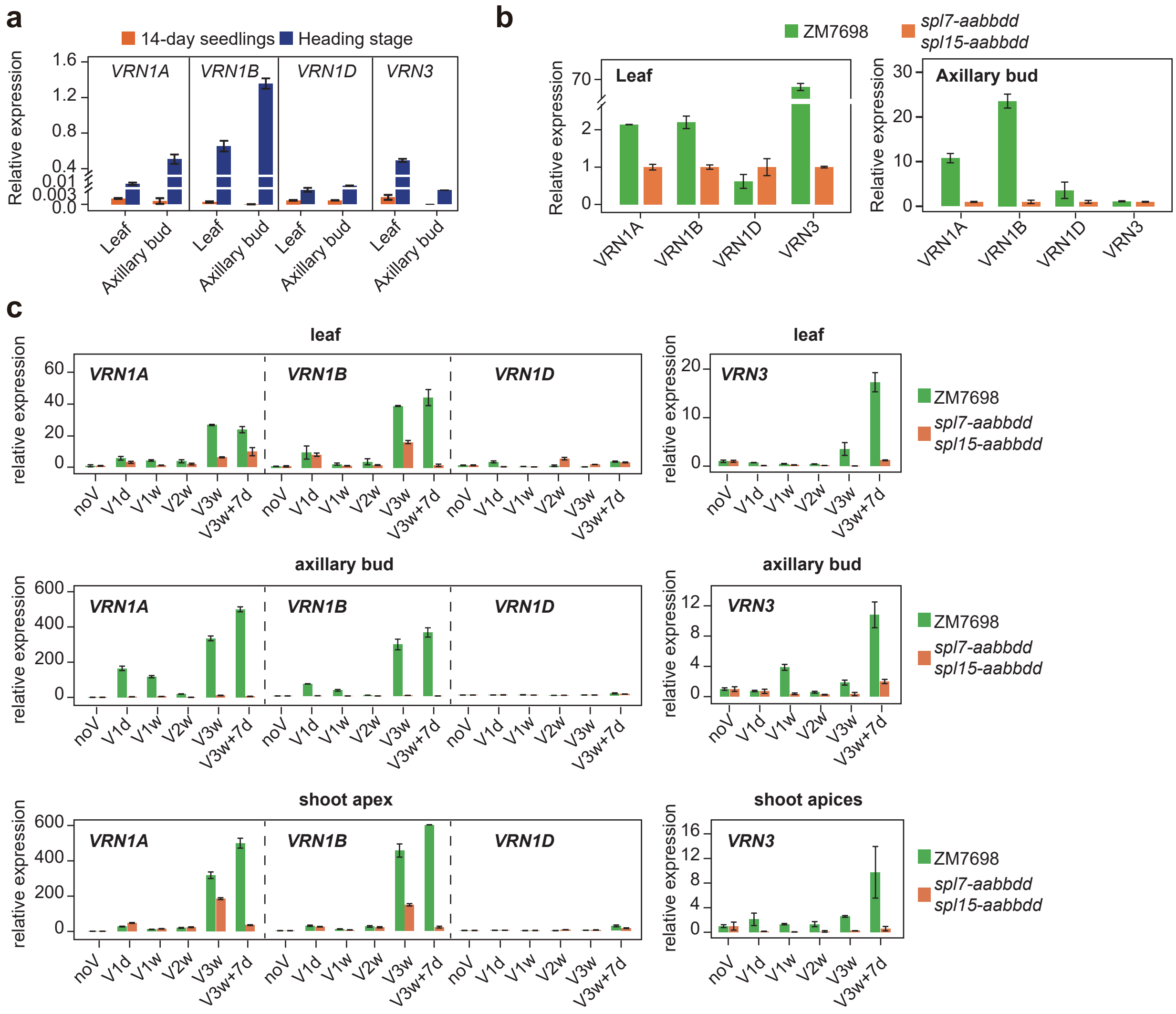

Fig. S10. Relative expression of *VRN1* and *VRN3* in leaf, axillary bud and shoot apices.
a-b. Relative expression of *VRN1* and *VRN3* in leaf and axillary bud. **a.** Expression of *VRN1* and *VRN3* were increased during development without vernalization. **b.** Up-regulation of *VRN1* and *VRN3* were repressed in *spl7-aabbdd spl15-aabbdd* hexa-mutant in un-vernalization conditions. Leaves and axillary buds from ZM7698 and *sspl7-aabbdd spl15-aabbdd* hexa-mutant were collected from 14-day old seedlings or plants 1 day after heading (no vernalization). **c.** Vernalization response of *VRN1* and *VRN3*. noV means plants growing in greenhouse (day for 16h at 22°C, night for 8h at 19°C) for 2 weeks. V1d means plants growing in greenhouse (day for 16h at 22°C, night for 8h at 19°C) for 2 weeks and vernalization (4°C, 16 h light/8 h dark) for 1 day. V1w means plants growing in greenhouse (day for 16h at 22°C, night for 8h at 19°C) for 2 weeks and vernalization (4°C, 16 h light/8 h dark) for 1 week, same for V2w and V3w. V3W+7d means plants growing in greenhouse (day for 16h at 22°C, night for 8h at 19°C) for 2 weeks, then vernalization (4°C, 16 h light/8 h dark) for 3 weeks and growing in greenhouse (day for 16h at 22°C, night for 8h at 19°C) for 7 days after vernalization (4°C, 16 h light/8 h dark).
