# Supplementary material for: Epigenomic identification of vernalization cis-regulatory elements in winter wheat
Source: Genome Biol. 2024 Jul 30;25:200. doi: 10.1186/s13059-024-03342-3 (PMC11290141; doi:10.1186/s13059-024-03342-3)
Supplement: Supplementary file 15 — Additional file 15. Review history. [file 13059_2024_3342_MOESM15_ESM.docx]

Review History

**First round of review**

**Reviewer 1 and 3**

**Were you able to assess all statistics in the manuscript, including the appropriateness of statistical tests used?**

Yes, and I have assessed the statistics in my report.

**Comments to author:**

Epigenetic regulation plays an important role in vernalization across in many plant species, including winter wheat. However, the key cis-regulatory elements and the molecular mechanism of vernalization in wheat have not been well studied. Liu et al. revealed that the global profile of H3K27ac, H3K4me3, and H3K27me3 in leaves, axillary buds, and shoot apices both before and after vernalization. Through integration with ATAT-seq and RNA-seq, the authors concluded that epigenetic modifications are associated with tissue-specific responses and sub-genome-divergent expression after vernalization. More important, they identified the enhancers associated with vernalization and discovered a vernalization-related enhancer VRN3. Combined the published DAP-seq data, they found that TaSPL7/15, which are aging-related components in flowering pathway, target the VRN1 promoter and VRN3 enhancer to regulate their expression. Overall, the findings are interesting. Below are some suggestions to improve the manuscript:

1. Scatter plots (Fig. S1) were used in the analysis of RNA-seq, ATAT-seq, and ChIP-seq to evaluate the reproducibility between two replicates for each sample. Avoiding the use of the count numbers is recommended due to the non-linearity of their logarithmic values. Instead, using the FPKM value is more accurate.

2. The IGV images for both replicates should be displayed in all figures.

3. The authors examined the correlations between induced and repressed Differentially Expressed Genes (iDEGs/rDEGs) and the four histone modifications (Fig. 2).

a. In 2b, there is no difference in H3K4me3 levels for repressed DEGs with or without vernalization in leaves. Therefore, the description "the levels of active histone modifications increased after vernalization for the induced DEGs, and decreased for the repressed DEGs" is not correct (Lines 165-167).

b. H3K27me3 is decreased in both induced DEGs and repressed DEGs. Given that H3K27me3 is associated with gene repression, and that the levels of H3K27me3 are much higher for iDEGs than those for rDEGs, the authors should further discuss this unexpected result. Might this indicate that H3K27me3 is not playing a major role in the control of expression of induced DEGs and repressed DEGs, or that other factors are more important in the lowered expression of the repressed DEGs?

c. The author claims that "However, the H3K27me3 level of the induced DEGs was much higher than that of the repressed DEGs, and H3K27me3 levels were reduced in both induced and repressed DEGs (Fig. 2b), implying a different regulating pattern from FLC in Arabidopsis." (Lines 167-170). It is not appropriate to compare genome-wide H3K27me3 changes in wheat with the alterations in a single gene in Arabidopsis. What would be appropriate is a comparison of genome-wide studies in wheat and Arabidopsis. Thus, the comment "implying a different regulating pattern from FLC in Arabidopsis" should be removed.

4. The putative promoters or enhancers located more than 2kb from the gene were characterized by the presence or absence of H3K4me3. However in the 2022 Genome Biology study by Xie. et al., the enhancers in wheat exhibit marking of both H3K27ac and H3K4me3. Furthermore, the IGV of VRN3 in Fig. 4c reveals a modest increase in H3K4me3 levels after vernalization within the open chromatin region based on ATAC-seq. Therefore, it would be important to provide a more detailed explanation of your definitions of the putative promoters and enhancers.

5. The author selected three representative genes (VRN1, VRN3, and WSOC1) to confirm the enhancer activity associated with vernalization. Despite the open chromatin structure of WSOC1, there is an increase in H3K27me3, while H3K27ac and H3K4me3 decrease after vernalization. Furthermore, vernalization promotes the activity of the WSOC1 enhancer. How can this be explained? Does it imply that the open chromatin state is not necessarily linked to the histone modification?

6. SPLs binding motifs are specifically enriched in the putative enhancers; thus, SPL5/17 was selected for detailed analysis. However, considering the overall expression pattern of all SPLs (Fig. S9a), the mRNA levels of SPL17 do not show a significant change before or after vernalization in the three tissues, as compared to other SPL genes, including SPL14, SPL4, and SPL2. In addition, based on the IGV analysis of DAP-seq data (Fig. 6c), SPL7A and SPL15B target the gene body region, showing a prominent peak around the first intron of VRN1. In the case of VRN3, the peak is located in the promoter region. The peak region induced by vernalization in ATAT-seq is more likely attributable to noise signals in DAP-seq.

7. In Figure 6g, the images of tobacco leaves show no signals for P4. However, SPLs can activate P4 fragments, as indicated by the LUC/REN value. Furthermore, the dual luciferase reporter assay should be conducted using the mutated elements within the target region as reporter because this would provide the evidence regarding whether SPL5/17 promotes transcription by binding the specific motif.

8. For Fig. 7j, it's better to show the flowering time rather than the percentage of flowering plants. In addition, it's necessary to include the leaf number. The delayed flowering phenotype in mutants may be attributed to slower growth, specifically a slower emergence of leaves. The number of leaves formed on the parent culm is a critical metric in the description of flowering phenotypes.

9. According to the model presented in Figure 7, SPLs target VRN1 to enhance its expression. VRN1 and VRN3 interact in a positive feedback loop. However, VRN1, but not VRN3, is induced during vernalization. Thus, the author should investigate the expression of VRN1 in both WT and SPL7/15 mutants during a time course of vernalizing cold treatment to elucidate the dynamic impact of SPL7/15 on VRN1 independent of VRN3.

Minor comments:

1. In Fig. 1b, only VRN is currently labeled for both genes. Correct this by specifically marking the VRN1 and VRN2 genes.

2. In the figure legends of Fig. 2d, "Red bars represent increased histone modifications after vernalization, green bars represent decreased" (Lines 581-582). However, there are no red or green bars in Fig. 2d.

3. The figure legends for Fig. 3a were duplicated in lines 596-602.

4. In Fig. 6d and 6e, SPL7A proteins are represented as SPL7A-GST and GST-SPL7A. Is the GST tag located individually at the C- or N-terminus?

5. Figure 7h lacks information regarding sample details, including the harvested time and growth stage. Additionally, only the sub-genome expression of VRN1 is presented, VRN3 should be also provided.

6. Line 641, need to add the corresponding figure number.

7. Need to add the figure legends of Fig. 7 to explain the model.

8. Lines 451 to 452, it's better to use the zeitgeber time instead of the precise time when harvesting the plants, since the readers lack information regarding the illumination period.

9. In line 495, the expression Log2(Fold change)>1 should be revised to Log2IFold changeI>1 or simply, Fold change >2.

10. The description of the methods is below the minimum needed for others to repeat the experiments, particularly in the case of EMSA (Lines 534 to 536).

**Reviewer 2**

**Were you able to assess all statistics in the manuscript, including the appropriateness of statistical tests used?**

No, I do not feel adequately qualified to assess the statistics.

**Comments to author:**

In this manuscript, the authors reported the epigenomic landscape before and after vernalization in winter wheat. They collected the mRNA-seq and ATAC-seq data from 3 different tissues before and after the vernalization, and the histone modifications data specifically for leaf samples before and after vernalization was collected too. By integrative analysis of the data, the author concluded that the vernalization response is quite different among different tissues. The H3K27me3 is more relevant to the vernalization-induced gene than the repressed genes. The authors also claimed that they have identified more than 10,000 enhancers, including one situated 30kb upstream of VRN3. The authors then followed this up and identified a transcription factor SPL7/15 that is able to bind this enhancer and regulate VRN3 expression. Finally, the author showed SPL7/15-VRN3/VRN1 form a regulatory module contributing to vernalization response. Overall, vernalization in winter wheat is undoubtedly an important subject, and comprehensive epigenomic profiling has not yet been published in a peer-reviewed journal. The manuscript provides a valuable resource to the vernalization research community and comes up with some interesting conclusions. With that said, there are many places in the manuscript that need further revision, and in some cases, thorough revision is required. Places related to enhancers identification and characterization are generally unconvincing. In addition, the current manuscript is quite difficult to read, so the overall writing and logic need to be polished to increase the accessibility to the general audience. Below I detail my concerns.

Major Concerns:

1. Related to Fig. 1c to f. the author showed the differential ACRs and DEGs are quite different among different tissues and concluded that vernalization responses of different tissues are dramatically different. More analysis is needed here. The gene expression pattern and ACRs before vernalization treatment of leaves, axillary buds, and shoot apices should already be very different. Therefore, how much of the vernalization-responsive ACRs and DEGs are associated with the differential gene expression pattern before vernalization should be considered and described.

2. Related to the point above, it is interesting that the number of DEGs and differential ACRs is much higher in leaves compared with the other two tissues. What does that mean? The author should briefly discuss this in the main text.

3. Line 163, the author claimed that chromatin accessibility around DEGs was generally correlated with the expression changes. However, I couldn't reach the same conclusion by looking at Fig. 2a. There is no significant decrease in chromatin accessibility for the repressed gene in the leaf (VL). Nevertheless, the chromatin accessibility data should be displayed in a better way such that it is more quantitative and comparable between samples.

4. In lines 167-170, Fig.2b, the author found that H3K27me3 levels of the induced DEGs were a lot higher than the repressed DEGs and mentioned that such a pattern is different from that of FLC in Arabidopsis. There are several issues here. First of all, such data needs to be carefully interpreted and more analysis is definitely needed here. The metagene plot displays the average of H3K27me3 over many genes. There are at least several different possibilities to match such data with totally different biological meanings. For example, the higher/lower H3K27me3 in the induced/repressed gene group could be due to a small subgroup of genes displaying particularly high or low H3K27me3 or due to a larger/smaller proportion of genes bearing H3K27me3. More analysis is needed to clarify these possibilities. In addition, the comparison with FLC is inappropriate and confusing, given the latter is a single gene with a defined pattern. It is very likely that there are examples in the repressed group exactly like FLC. I also noticed that both the induced and the repressed DEGs showed a decrease of H3K27me3 after vernalization (Fig. 2b). The H3K27me3 is decoupled from the expression for the repressed DEGs; what's the explanation for that?

5. It is almost impossible to understand Fig.2f (Line 190). What is ChromHMM? What are the 9 states? What are the Emissions? I can't find any information in either figure legend/results/methods.

6. Fig. 2g and the associated results are quite interesting. I noticed that a larger proportion of the "mixed" group was altered compared with the other states. What is the nature of the mixed state? Why are they more responsive to vernalization than the other groups? More description and discussion of the mixed states is needed in the main text.

7. Line 234 to Line 236, the author claimed that chromatin accessibility, H3K36me3, and H3K27ac contributed the most to unbalanced expression changes. I am not quite sure how such a conclusion is reached. For example, what's the difference between H3K4me3 and H3K36me3? What is the evidence that histone marks are the driver of the subgenome-specific responses? Nevertheless, a careful description of the Fig.3g as well as the underlying logic are missing here.

8. Line 242 to Line 264, the authors described the identification of vernalization-related enhancers. There are several issues related to this part. Apparently, the word "enhancer" has a definitive meaning and should not be generalized as in the current manuscript. The author found many putative distal regulatory ACRs instead of enhancers. Enhancer means a distal element that is able to interact with the promoter; thus, without such data, the word enhancer should not be used.

Similarly, in Fig. 4f and g, the author examined the GWAS signals of certain traits within 1kb of identified regulatory ACRs. Although the data do support those regulatory elements are generally meaningful, there is no data to support so-called enhancers are correct, a comparison of enhancers versus non-enhancers but with similar chromatin accessibility is missing here. In other words, regulatory ACRs proximal or distal to the gene are linked with traits, unsurprisingly, but no claim can be made about whether they are enhancers.

The authors performed a LUC assay to validate the enhancers in Fig 4i. I found this assay problematic. The assay just simply puts an ACR immediately before a mini-promoter. The spatial information is lost in this case. Is there any negative control that a certain ACR can not activate the transcription of the reporter? I doubt that.

9. The author argued that the SPL7/15 regulates vernalization, yet the flowering time phenotype of the mutant is mainly before vernalization. This seems to argue that these genes mainly do not function during vernalization. Nevertheless, more discussion is needed at least.

Minor concerns:

10. Genome browser tracks of individual repeats should be displayed to demonstrate the reproducibility of the data better.

11. Line 54，"cold signal triggers SAM transition to reproductive SAM…," a reference is needed here.

12. Line 57, the nature of VRN2 protein should be briefly introduced.

13. Line 70, there should be no H3K9 at FLC; the results from some early-year publications might not be accurate. please remove such a claim.

14. Related to Fig1b and Fig. S2, what's the difference among VRN1A/B/D in terms of histone modifications before and after vernalization? This should be described and displayed at least as supplementary data.

15. Line 210-212, citations are needed here.

16. Line 217 to Line 219, examples of different modes of subgenome-specific vernalization responses should be displayed, at least in the supplementary data.

17. Line 283-286, the connection with the previous paragraph is quite odd. Please consider re-writing this part.

18. Figure 6f and g, what is P5? Figure 6g: where are the results of P2? Figure 4i, what are the P1 to P5? The information or results are missing and should be added.

19. Please carefully update the figure legends. Currently, it is difficult to read as much information was omitted. Many abbreviations were not properly defined.

**Authors Response**

**Point-by-point responses to the reviewers’ comments:**

**Reviewer #1&3:**

Epigenetic regulation plays an important role in vernalization across in many plant species, including winter wheat. However, the key cis-regulatory elements and the molecular mechanism of vernalization in wheat have not been well studied. Liu et al. revealed that the global profile of H3K27ac, H3K4me3, and H3K27me3 in leaves, axillary buds, and shoot apices both before and after vernalization. Through integration with ATAT-seq and RNA-seq, the authors concluded that epigenetic modifications are associated with tissue-specific responses and sub-genome-divergent expression after vernalization. More important, they identified the enhancers associated with vernalization and discovered a vernalization-related enhancer VRN3. Combined the published DAP-seq data, they found that TaSPL7/15, which are aging-related components in flowering pathway, target the VRN1 promoter and VRN3 enhancer to regulate their expression. Overall, the findings are interesting. Below are some suggestions to improve the manuscript:

***Response:*** *We appreciate your comments.*

1. Scatter plots (Fig. S1) were used in the analysis of RNA-seq, ATAT-seq, and ChIP-seq to evaluate the reproducibility between two replicates for each sample. Avoiding the use of the count numbers is recommended due to the non-linearity of their logarithmic values. Instead, using the FPKM value is more accurate.

***Response:*** *Thank you for your suggestion. We have replaced all analyses in Fig. S1 with FPKM in the revised version. Additionally, we supplemented the PCA for RNA-seq and ATAC-seq (Figs. S1d and S1e).*

*Figure for response (Fig. S1d and Fig. S1e of the revised version). PCA for ATAC-seq and RNA-seq. L means leaf, VL means vernalization leaf, AB means axillary bud, VA means vernalization axillary buds, SA means shoot apices, and VS means vernalization shoot apices.*

2. The IGV images for both replicates should be displayed in all figures.

***Response:*** *Thank you for your suggestion. We have added the tracks of two duplicates in all IGV screenshots, including Fig. 1b, Fig. 2h, Fig. 4c, Fig. 5f, Fig. 6c, Fig. S2a, Fig. S5c, Fig. S 6a, and Fig. S8c.*

3. The authors examined the correlations between induced and repressed Differentially Expressed Genes (iDEGs/rDEGs) and the four histone modifications (Fig. 2).

a. In 2b, there is no difference in H3K4me3 levels for repressed DEGs with or without vernalization in leaves. Therefore, the description "the levels of active histone modifications increased after vernalization for the induced DEGs, and decreased for the repressed DEGs" is not correct (Lines 165-167).

***Response:*** *Thank you for your suggestions. We have made changes in the revised version (PAGE 8, LINE 186): “The levels of active histone modifications (H3K4me3, H3K27ac and H3K36me3) increased after vernalization for the induced DEGs, and levels of H3K27ac and H3K36me3 decreased for the repressed DEGs”.*

b. H3K27me3 is decreased in both induced DEGs and repressed DEGs. Given that H3K27me3 is associated with gene repression, and that the levels of H3K27me3 are much higher for iDEGs than those for rDEGs, the authors should further discuss this unexpected result. Might this indicate that H3K27me3 is not playing a major role in the control of expression of induced DEGs and repressed DEGs, or that other factors are more important in the lowered expression of the repressed DEGs?

***Response:*** *Thank you for your professional comments. Combining your comments and those of another reviewer, we have reorganized this part. Previously, we profiled the levels of histone modifications on all up- and down-regulated genes. Now, we only examined the changes in histone modifications on differential expressed genes that have this type of modification in at least one tissues.*

*To explore why H3K27me3 levels are higher in iDEGs than in rDEGs, we characterized the modification levels of genes with H3K27me3 modification in iDEGs and rDEGs. 2,152 and 1,212 genes were highly enriched with H3K27me3 modification in iDEGs and rDEGs which accounts for 12.38% and 9.18% of total i/rDEGs, respectively. We then check the levels of H3K27me3 around those H3K27me3 modified DEGs, which showed* *H3K27me3 were decreased in the upstream and downstream regions of iDEGs, while only slightly increased in genebodies of rDEGs during cold vernalization (Fig. 2b). The result indicates that a decrease in H3K27me3 is likely associated with a higher percentage of iDEGs, while an increase in H3K27me3 is likely associated with a small subset of rDEGs. This is consistent with the observation that over 27.3% of the genes whose H3K27me3 levels decreased after vernalization were induced, whereas 5.8% of the genes whose H3K27me3 levels increased were repressed (Fig. 2d). The relative content were modified accordingly.*

*Figure for response (Fig. S5c of the revised version). The proportion of H3K27me3-modified genes among up-regulated and down-regulated genes. To determine whether the genes is modified by H3K27me3, the H3K27me3 ChIP-seq reads from 2kb upstream to 2kb downstream of genes were used to make a matrix and k-mean clustering were used to identify genes with higher read densities. iDEGs means up-regulated genes, and rDEGs means down-regulated genes during vernalization.*

*Figure for response. The H3K27me3 level of all DEGs (left, Fig. 2b of original version and Fig. S5b of the revised version) and DEGs bearing H3K27me3 (right, Fig. 2b of the revised version).*

c. The author claims that "However, the H3K27me3 level of the induced DEGs was much higher than that of the repressed DEGs, and H3K27me3 levels were reduced in both induced and repressed DEGs (Fig. 2b), implying a different regulating pattern from FLC in Arabidopsis." (Lines 167-170). It is not appropriate to compare genome-wide H3K27me3 changes in wheat with the alterations in a single gene in Arabidopsis. What would be appropriate is a comparison of genome-wide studies in wheat and Arabidopsis. Thus, the comment "implying a different regulating pattern from FLC in Arabidopsis" should be removed.

***Response:*** *Thank you for your suggestion. We agree with you and have removed the sentence in the revised version.*

4. The putative promoters or enhancers located more than 2kb from the gene were characterized by the presence or absence of H3K4me3. However in the 2022 Genome Biology study by Xie. et al., the enhancers in wheat exhibit marking of both H3K27ac and H3K4me3. Furthermore, the IGV of VRN3 in Fig. 4c reveals a modest increase in H3K4me3 levels after vernalization within the open chromatin region based on ATAC-seq. Therefore, it would be important to provide a more detailed explanation of your definitions of the putative promoters and enhancers.

***Response:*** *Thank you for your comments. H3K4me3 is usually considered to be the mark of promoter, and some other studies also believe that H3K4me3 co-localizes with enhancer (Shlyueva D, et al: Transcriptional enhancers: from properties to genome-wide predictions. Nat Rev Genet 2014, 15:272-286; Ricci WA, et al: Widespread long-range cis-regulatory elements in the maize genome. Nat Plants 2019, 5:1237-1249; Xie YL, et al: Enhancer transcription detected in the nascent transcriptomic landscape of bread wheat. Genome Biol 2022, 23). To accurately delineate the components and prevent disputes, we separately discussed the distal ACRs based on the presence or absence of H3K4me3, refraining from using the term 'enhancers' and instead opting for 'distal differential ACRs' in the revised version.*

5. The author selected three representative genes (VRN1, VRN3, and WSOC1) to confirm the enhancer activity associated with vernalization. Despite the open chromatin structure of WSOC1, there is an increase in H3K27me3, while H3K27ac and H3K4me3 decrease after vernalization. Furthermore, vernalization promotes the activity of the WSOC1 enhancer. How can this be explained? Does it imply that the open chromatin state is not necessarily linked to the histone modification?

***Response:*** *Thank you for your comments. In general, active histone modifications (H3K27ac, H3K36me3 and H3K4me3) are active marks which is usually associated with open chromatins, while regions neighboring with repressive histone (H3K27me3) is less accessible (Cai YC, et al: H3K27me3-rich genomic regions can function as silencers to repress gene expression via chromatin interactions. Nat Commun 2021, 12. 1.	Stepniak K, et al: Mapping chromatin accessibility and active regulatory elements reveals pathological mechanisms in human gliomas. Nat Commun 2021, 12.). To further confirm the general patterns in wheat, we examined the accessibilities of genes with and without these histone modifications across the genome. Genes with activating histone modifications have higher promoter accessibilities, while genes with* *repressive histone modifications have lower accessibilities (as shown below). Regarding WSOC1, we speculate that this discrepancy might arise from the diverse response patterns of various cell types, as our bulk assays can only capture changes in cell mixtures.*

*Figure for response (Fig. S5a of the revised version). We classified all genes in the whole genome according to the presence or absence of modifications, and screened the openness of the two categories of genes. H3K27ac represents the gene modified by H3K27ac, and noH3K27ac represents genes not modified, and the same applies to other modifications. The accessibilities of genes were characterized by the number of Tn5 insertion sites in ATAC-seq data.*

6. SPLs binding motifs are specifically enriched in the putative enhancers; thus, SPL5/17 was selected for detailed analysis. However, considering the overall expression pattern of all SPLs (Fig. S9a), the mRNA levels of SPL17 do not show a significant change before or after vernalization in the three tissues, as compared to other SPL genes, including SPL14, SPL4, and SPL2. In addition, based on the IGV analysis of DAP-seq data (Fig. 6c), SPL7A and SPL15B target the gene body region, showing a prominent peak around the first intron of VRN1. In the case of VRN3, the peak is located in the promoter region. The peak region induced by vernalization in ATAT-seq is more likely attributable to noise signals in DAP-seq.

***Response:*** *thank you for your comments. We agree with you that the expression changes of SPL7 and SPL15 are significant in specific tissues while not more severe than those of SPL14, SPL4 and SPL2. We choose SPL7 and SPL15 for further analysis based on the following reasons. TaSPL7/15 are the orthologous genes of Arabidopsis SPL9 and rice OsSPL14/IPA1, which has been proved to be important for age dependent flowering regulation and plant architecture regulation (Wang JW, et al: miR156-regulated SPL transcription factors define an endogenous* *flowering pathway in Arabidopsis thaliana. Cell 2009, 138:738-749; Jiao YQ, et al: Regulation of OsSPL14 by OsmiR156 defines ideal plant architecture in rice. Nat Genet 2010, 42:541–544). It’s also reported TaSPL7/15 related to wheat tillering and panicle development (Pei H, et al: Low-affinity SPL binding sites contribute to subgenome expression divergence in allohexaploid wheat. Sci China Life Sci 2023, 66:819-834), which are critical for wheat yields. Therefore, we think it's worth to check whether these genes are also involved in vernalization regulation.*

*For the DAP-seq parts, we agree with you that the highest peak around VRN1 is located within the first intron and the peak signal was not very high in VRN3 promoters. Although, we have further verified the interaction with EMSA and luciferase assays (Fig. 6d, Fig. 6e, Fig. 6g), we also repeated the DAP assays and use qPCR to further valid the enrichments of those regions (Figure below), which revealed significantly higher binding signals compared to the nearby regions.*

*Figure for response (Fig. S8f of the revised version). Quantification of peaks using DAP-qPCR. P1-P4 were designed according to the regions marked in Fig. 6c, and P5 was marked in Fig. S8c.*

7. In Figure 6g, the images of tobacco leaves show no signals for P4. However, SPLs can activate P4 fragments, as indicated by the LUC/REN value. Furthermore, the dual luciferase reporter assay should be conducted using the mutated elements within the target region as reporter because this would provide the evidence regarding whether SPL5/17 promotes transcription by binding the specific motif.

***Response:*** *Thank you for your comments. If looking at the y-axis, you would find that the signal of P4 is relatively low, more than one order of magnitude lower than the other probes. And the signal is too low to be detected by our instrument (NightSHADE evo LB 985N and* *PerkinElmer IVIS Lumina LT Series III). As you suggested, we supplemented experiments with fragments with mutated motifs, which showed that the signals of mutated elements were significantly weaker or almost lost compared with the wild type, indicating the specific binding of TaSPL7/15.*

*Figure for response (Fig. S8h of the revised version). Validation of TaSPL7/15’s* *specific binding to probes. P1 and P3 are the ACRs marked in Fig. 6c, and P5 is the ACR marked in Fig. S8c. Pn mut represents the Pn fragment with GTAC motif(s) mutated.*

8. For Fig. 7j, it's better to show the flowering time rather than the percentage of flowering plants. In addition, it's necessary to include the leaf number. The delayed flowering phenotype in mutants may be attributed to slower growth, specifically a slower emergence of leaves. The number of leaves formed on the parent culm is a critical metric in the description of flowering phenotypes.

***Response:*** *Thank you for your suggestions. Following your suggestion, we changed the percentage of flowering plants in Fig. 6j to the flowering time. According to your suggestions, we counted the number of total leaves, which showed that the leaf counts in spl7aabbdd spl15aabbdd hexa-mutants were much more than that of the wild type before heading (Fig. S9b and Fig. S9c). The result* *is similar with the more rosette leaves and late flowering phenotype of spl9 mutants in Arabidopsis (Wang JW, et al: miR156-regulated SPL transcription factors define an endogenous flowering pathway in Arabidopsis thaliana. Cell 2009, 138:738-749).*

Figure for response (Fig. 6j of the revised version). Statistics of heading time.

*Figure for response (Fig. S9b (left) and Fig. S9c (right) of the revised version). Leaf number of ZM7698 and spl7-aabbdd spl15-aabbdd.*

9. According to the model presented in Figure 7, SPLs target VRN1 to enhance its expression. VRN1 and VRN3 interact in a positive feedback loop. However, VRN1, but not VRN3, is induced during vernalization. Thus, the author should investigate the expression of VRN1 in both WT and SPL7/15 mutants during a time course of vernalizing cold treatment to elucidate the dynamic impact of SPL7/15 on VRN1 independent of VRN3.

***Response:*** *Thank you for your comments. As you suggested, we investigated the expression of VRN1 during a time course of vernalization, including 14 days seedlings, 1 day cold treatment, 1 week, 2 weeks and 3 weeks cold treatment, 1 day in green house after 3 weeks cold treatment and 7 days in green house after 3 weeks cold treatment (Fig. S10c of the revised version).* *It was observed that the fold changes of VRN1 in the spl7-aabbdd spl15-aabbdd hexa-mutants during vernalization were much lower than those in the wild type (as depicted in the following figure). Additionally, VRN3 hardly responded during the vernalization process and only began to show a response after vernalization was completed. These findings suggest that the* *repression of VRN1's response in spl7-aabbdd spl15-aabbdd hexa-mutants precedes that of VRN3.*

*Figure for response (Fig. S10c of the revised version). Vernalization response of VRN1 and VRN3. noV means plants grow in greenhouse for 2 weeks. V1d means plants grow in greenhouse for 2 weeks and cold treatment for 1 day. V1w means plants grow in greenhouse for 2 weeks and cold treatment for 1 week, same for V2w and V3w. V3W+7d means plants grow in greenhouse for 2 weeks, then cold treatment for 3 weeks and grow in greenhouse for 7 days after cold treatment.*

Minor comments:

1. In Fig. 1b, only VRN is currently labeled for both genes. Correct this by specifically marking the VRN1 and VRN2 genes.

***Response:*** *Thank you for your suggestions. We have corrected them in Fig. 1b.*

2. In the figure legends of Fig. 2d, "Red bars represent increased histone modifications after vernalization, green bars represent decreased" (Lines 581-582). However, there are no red or green bars in Fig. 2d.

***Response:*** *Thank you for your comments. We carefully checked Fig. 2d and the text, and* *corrected the colors in Fig. 2d.*

3. The figure legends for Fig. 3a were duplicated in lines 596-602.

***Response:*** *Thank you for your suggestion. We have deleted the duplicate legends.*

4. In Fig. 6d and 6e, SPL7A proteins are represented as SPL7A-GST and GST-SPL7A. Is the GST tag located individually at the C- or N-terminus?

***Response:*** *Thank you for bringing this to our attention. The indication regarding the individual localization of GST at the C- or N-terminus was incorrect. This mislabeling has been rectified, and the accurate designation, GST-SPL7A, has been applied in Fig. 6e.*

5. Figure 7h lacks information regarding sample details, including the harvested time and growth stage. Additionally, only the sub-genome expression of VRN1 is presented, VRN3 should be also provided.

***Response:*** *Thank you for your comments. The specific information about RT-qPCR sample details has been listed in Methods (PAGE 25, LINE 588-593), and we have added detail information about the growth period and sampling in the Figure legend of the revised version. The updated legend states: “Samples for RT-qPCR were obtained from plants grown 2 weeks (day for 16h at 22°C, night for 8h at 19°C) after germination and 7 days in greenhouse (day for 16h at 22°C, night for 8h at 19°C) after vernalization (plants grown 2 weeks in green house and vernalization for 3 weeks). All samples were taken between 9AM and 12AM (China Standard Time).”*

*We agree with your comments. We have designed several primer pairs to distinguish the sub-genome copies of VRN3 following your suggestions. However, due to the high sequence similarity of the three gene copies, we ultimately failed to find a sub-genome specific primer pairs for the qPCR.*

6. Line 641, need to add the corresponding figure number.

***Response:*** *thank you for your comment. We have added the corresponding figure number in figure legend of Fig. 4.*

7. Need to add the figure legends of Fig. 7 to explain the model.

***Response:*** *Thank you for your suggestion. We have added the figure legends of Fig. 7 to explain the model. “**During the vernalization process in wheat, the expression of VRN1 and VRN3 increases, while the expression of VRN2 decreases. Concurrently, the modifications of* *H3K27ac, H3K36me3, and H3K4me3 on VRN1 increase, whereas the level of H3K27me3 modification around VRN1 decreases. The modifications of H3K36me3 and H3K4me3 on VRN3 also increase, along with an increase in H3K27ac levels on the distal regulatory elements, while the surrounding H3K27me3 levels decrease. Numerous transcription factors are involved in the regulation of vernalization, among which SPL7 and SPL15 are induced by vernalization. These factors regulate wheat vernalization by directly binding to the VRN1 promoter and VRN3 distal regulatory elements, thereby activating their expression in leaves and apical meristems.”*

8. Lines 451 to 452, it's better to use the zeitgeber time instead of the precise time when harvesting the plants, since the readers lack information regarding the illumination period.

***Response:*** *Thank you for your suggestion. We have added zeitgeber time in Methods section (PAGE 22, LINE 506-509): “All samples, including leaves, axillary buds, and shoot apices, were taken between 9 AM and 12 PM (China Standard Time), which corresponds to 1-3 hours after the start of the light cycle (our light cycle is from 8 AM to 12 AM, and the dark cycle is from 12 AM to 8 AM)”.*

9. In line 495, the expression Log2(Fold change)>1 should be revised to Log2IFold changeI>1 or simply, Fold change >2.

***Response:*** *Thank you for your suggestion. We have replaced Log2(Fold change)>1 with Fold change >2.*

10. The description of the methods is below the minimum needed for others to repeat the experiments, particularly in the case of EMSA (Lines 534 to 536).

***Response:*** *Thank you for your suggestion. We have reviewed and supplemented methods accordingly.*

**Reviewer #2:**

In this manuscript, the authors reported the epigenomic landscape before and after vernalization in winter wheat. They collected the mRNA-seq and ATAC-seq data from 3 different tissues before and after the vernalization, and the histone modifications data specifically for leaf samples before and after vernalization was collected too. By integrative analysis of the data, the author concluded that the vernalization response is quite different among different tissues. The H3K27me3 is more relevant to the vernalization-induced gene than the repressed genes. The authors also claimed that they have identified more than 10,000 enhancers, including one situated 30kb upstream of VRN3. The authors then followed this up and identified a transcription factor SPL7/15 that is able to bind this enhancer and regulate VRN3 expression. Finally, the author showed SPL7/15-VRN3/VRN1 form a regulatory module contributing to vernalization response. Overall, vernalization in winter wheat is undoubtedly an important subject, and comprehensive epigenomic profiling has not yet been published in a peer-reviewed journal. The manuscript provides a valuable resource to the vernalization research community and comes up with some interesting conclusions. With that said, there are many places in the manuscript that need further revision, and in some cases, thorough revision is required. Places related to enhancers identification and characterization are generally unconvincing. In addition, the current manuscript is quite difficult to read, so the overall writing and logic need to be polished to increase the accessibility to the general audience. Below I detail my concerns.

***Response:*** *We appreciate your comments.*

Major Concerns:

1. Related to Fig. 1c to f. the author showed the differential ACRs and DEGs are quite different among different tissues and concluded that vernalization responses of different tissues are dramatically different. More analysis is needed here. The gene expression pattern and ACRs before vernalization treatment of leaves, axillary buds, and shoot apices should already be very different. Therefore, how much of the vernalization-responsive ACRs and DEGs are associated with the differential gene expression pattern before vernalization should be considered and described.

***Response:*** *Thank you for your comments. We further identified the tissue-specific expressed genes by pairwise comparison and then profiled their pattern changes. Take the compartment between leaves and axillary buds as an example, more than half of genes were kept in the same groups during vernalization. Then similar results were found with vernalization-responsive genes and differential ACRs, which also suggested different responses among different tissues during vernalization. Notably, the conversion percentage of responsive genes and ACRs were higher than the total expressed genes, especially when comparing SA with other tissues, indicating tissue specific expression are important during vernalization responses.*

*Figure for response (Fig. S4a of the revised version). The tissue specific expression patterns of all expressed genes. The three pictures are the results of comparison between each two tissue pairs, and the conditions before and after vernalization have been identified. L means genes highly expressed in leaf. AB means genes highly expressed in axillary buds. SA means genes highly expressed in shoot apices. Co means genes that are not differentially expressed between two tissues. Differentially expressed genes (DEGs) were identified using DESeq2 with Foldchange > 2 and p-value < 0.01. Here we count all expressed genes, that is, genes whose TPM > 1 in at least one tissues before and after vernalization.*

*Figure for response (Fig. S4b of the revised version). The tissue specific expression pattern of vernalization-responsive DEGs. All these DEGs are part of the expressed genes in picture above.*

*Table for response (Table. S2 of the revised version). The proportion of vernalization-responsive genes and diffACR-related vernalization-responsive genes among genes with altered tissue expression patterns. L vs AB means comparison between leaf and axillary bud. L vs SA means comparison between leaf and shoot apices. AB vs SA means comparison between axillary bud and shoot apices. total represents genes highly expression in the first tissues before vernalization. shift DEGs means genes whose tissue expression pattern changed before and after vernalization and the genes were also vernalization-responsive gene. diffACR related means genes that are diffACRs related to shift DEGs.*

2. Related to the point above, it is interesting that the number of DEGs and differential ACRs is much higher in leaves compared with the other two tissues. What does that mean? The author should briefly discuss this in the main text.

***Response:*** *Thank you for your comment. We hypothesize that the more pronounced response to low-temperature vernalization in leaves is attributed to their greater cellular diversity and relatively heightened exposure to environmental stimuli compared to axillary buds and apical meristems, which are enveloped within multiple layers of leaves. And we have added descriptions in the main text “Interestingly, the vernalization response of leaves was greater than that of axillary buds and shoot apices, which was reflected by the counts of differential ACRs and DEGs. It may attribute to their greater cellular diversity and relatively heightened exposure to environmental stimuli compared to axillary buds and apical meristems, which are enveloped within multiple layers of leaves” (PAGE 8, LINE 175-179).*

3. Line 163, the author claimed that chromatin accessibility around DEGs was generally correlated with the expression changes. However, I couldn't reach the same conclusion by looking at Fig. 2a. There is no significant decrease in chromatin accessibility for the repressed gene in the leaf (VL). Nevertheless, the chromatin accessibility data should be displayed in a better way such that it is more quantitative and comparable between samples.

***Response:*** *Thank you for your comments. To further quantify the chromatin accessibility changes during vernalization, we performed statistics on the chromatin accessibility of DEGs in different tissues. The results revealed that, for the up-regulated DEGs of leaf and axillary bud, the chromatin accessibilities were increased, while for the down-regulated DEGs of leaves, axillary bud and shoot apices, the chromatin accessibilities were decreased (Fig. S5a of the revised version).*

*Figure for response (Fig S. 5a of the revised version). Chromatin accessibility of differentially expressed genes in different tissues. Chromatin accessibility was characterized by the number of Tn5 insertion sites, which was normalized between different samples by the size of the ATAC-seq library. The ordinate count represents the number of insertion sites after normalization. up means up-regulated DEGs, and down means down-regulated DEGs.*

4. In lines 167-170, Fig.2b, the author found that H3K27me3 levels of the induced DEGs were a lot higher than the repressed DEGs and mentioned that such a pattern is different from that of FLC in Arabidopsis. There are several issues here. First of all, such data needs to be carefully interpreted and more analysis is definitely needed here. The metagene plot displays the average of H3K27me3 over many genes. There are at least several different possibilities to match such data with totally different biological meanings. For example, the higher/lower H3K27me3 in the induced/repressed gene group could be due to a small subgroup of genes displaying particularly high or low H3K27me3 or due to a larger/smaller proportion of genes bearing H3K27me3. More analysis is needed to clarify these possibilities. In addition, the comparison with FLC is inappropriate and confusing, given the latter is a single gene with a defined pattern. It is very likely that there are examples in the repressed group exactly like FLC. I also noticed that both the induced and the repressed DEGs showed a decrease of H3K27me3 after vernalization (Fig. 2b). The H3K27me3 is decoupled from the expression for the repressed DEGs; what's the explanation for that?

***Response:*** *Thank you for your professional comments. Combining your comments and those of another reviewer, we have reorganized this part. Previously, we profiled the levels of histone modifications on all up- and down-regulated genes. Now, we added plots of histone modification levels on genes enriched with those markers in at least one tissues among up- and down-regulated genes.*

*To explore why H3K27me3 levels are higher in iDEGs than in rDEGs, we characterized the modification levels of genes with H3K27me3 modification in iDEGs and rDEGs. 2,152 and 1,212 genes were highly enriched with H3K27me3 modification in iDEGs and rDEGs which accounts for 12.38% and 9.18% of total i/rDEGs, respectively (pie chart below). We then check the levels of H3K27me3 around those H3K27me3 modified DEGs, which showed H3K27me3 were slightly decreased in the genebodies of iDEGs while slightly increased in upstream regions of rDEGs during cold vernalization (Fig. 2b), which is consistent with the repressing roles of H3K27me3. These results suggest that H3K27me3 is closely associated with changes in a small subset of genes, although its proportion is not as high as that of other types of modifications. But this does not indicate H3K27me3 were not important for repressed expressions, as more than 27.3% of the genes whose H3K27me3 levels increased after vernalization were down-regulated (Fig. 2d). The relative contents were modified accordingly.*

*Figure for response (Fig. S5c of the revised version). The proportion of H3K27me3-modified genes among up-regulated and down-regulated genes. The blue part in the pie chart represents the proportion of H3K27me3 modified genes. iDEGs means up-regulated genes, and rDEGs means down-regulated genes.*

*Figure for response. The H3K27me3 level of all DEGs (left, Fig. 2b of original version and Fig. S5b of of the revised version) and DEGs bearing H3K27me3 (right, Fig. 2b of the revised version).*

*Following your reminder, we realized that the comparison between the overall level and the single FLC gene was not appropriate, so we have deleted these descriptions.*

5.It is almost impossible to understand Fig.2f (Line 190). What is ChromHMM? What are the 9 states? What are the Emissions? I can't find any information in either figure legend/results/methods.

***Response:*** *Thank you for your comments. ChromHMM is a chromatin state annotation software based on multivariate hidden Markov model (HMM).* *And the 9 states are 9 chromatin states with different chromatin accessibility degree and histone modifications, arranged vertically in 9 rows (Fig. 2f). The Emissions are horizontal representation of chromatin accessibilities and different histone modifications. And we added relevant descriptions in the main text “To further understand the transition of chromatin states during vernalization, we classified chromatin into 9 states using ChromHMM, a chromatin state annotation software based on multivariate hidden Markov model (HMM) [38,39] (Fig. 2f). Each of the 9 chromatin states has varying degrees of chromatin accessibility and histone modifications”.*

6. Fig. 2g and the associated results are quite interesting. I noticed that a larger proportion of the "mixed" group was altered compared with the other states. What is the nature of the mixed state? Why are they more responsive to vernalization than the other groups? More description and discussion of the mixed states is needed in the main text.

***Response:*** *Thank you for your comments. We believe there are two scenarios to explain the occurrence of the "mixed" group phenomenon. Firstly, the samples used for these assays consist of bulk populations containing a mixture of different cells. It is plausible that certain genomic* *regions exhibit an activated state in some cells while being repressed in others within the same sample. This heterogeneity in cellular states could arise due to variations in cellular composition or treatment conditions. Consequently, these regions are more prone to exhibiting variability among cells or treatments, potentially undergoing transitions between active and repressed states (Altschuler SJ, et al: cellular heterogeneity: do differences make a difference? Cell 2010, 141:559-563; Vastenhouw NL et al: Bivalent histone modifications in early embryogenesis. Current Opinion in Cell Biology 2012, 24:374-386). Secondly, the presence of bivalent modifications could contribute to the "mixed" group phenomenon. Bivalent modifications refer to genomic regions marked by both activating and repressive histone modifications. These regions may display characteristics of both active and repressed chromatin states, leading to ambiguity in their functional status. The existence of bivalent modifications could result in variability in gene expression or chromatin accessibility across different cells or conditions (Li FF, et al: bivalent histone modifications and development. Curr Stem Cell Res T 2018, 13:83-90; Vastenhouw NL, et al: Bivalent histone modifications in early embryogenesis. Current Opinion in Cell Biology 2012, 24:374-386.; Zeng ZX, et al: Cold stress induces enhanced chromatin accessibility and bivalent histone modifications H3K4me3 and H3K27me3 of active genes in potato. Genome Biol 2019, 20.). In summary, these two scenarios provide potential explanations for the observed heterogeneity in the "mixed" group, highlighting the complex interplay between cellular heterogeneity and dynamic chromatin states in gene regulation. We also added those in the main text (PAGE 10, LINE 230-232) accordingly.*

7. Line 234 to Line 236, the author claimed that chromatin accessibility, H3K36me3, and H3K27ac contributed the most to unbalanced expression changes. I am not quite sure how such a conclusion is reached. For example, what's the difference between H3K4me3 and H3K36me3? What is the evidence that histone marks are the driver of the subgenome-specific responses? Nevertheless, a careful description of the Fig.3g as well as the underlying logic are missing here.

***Response:*** *Thank you for your comments. We evaluated the impact of epigenetic modifications on subgenom-unbalanced responses during vernalization by examining the correlation between subgenome responses and epigenetic responses (chromatin accessibility and histone* *modifications) during vernalization. It can be seen from the figure (Fig. 3g) that 48.66% of chromatin accessibility, 31.46% of H3K27ac, 26.93% of H3K36me3 and 23.43% of H3K4me3 subgenome unbalanced responses are related to the subgenome unbalanced responses of gene expression during vernalization, indicating that chromatin accessibility, H3K27ac, H3K36me3 and H3K4me3 show greater influence. In this instance, there are no obvious differences between H3K4me3 and H3K36me3. Previous errors in descriptions and numerical statistics have been corrected in the images (Fig. 3e and Fig. 3g) and text (“By calculating the overlap between various epigenetic modification responses and gene expression responses, we discovered that chromatin accessibility, H3K27ac, H3K36me3, and H3K4me3 were more closely linked with unbalanced expression changes”).*

*As your pointed, we can only see a correlation between the unbalanced subgenome responses of epigenetic modification and unbalanced response of gene expression, which cannot prove that histone marks are the driver of the subgenome-specific responses. Therefore, we modified the descriptions of this part according to your suggestions.*

8. Line 242 to Line 264, the authors described the identification of vernalization-related enhancers. There are several issues related to this part. Apparently, the word "enhancer" has a definitive meaning and should not be generalized as in the current manuscript. The author found many putative distal regulatory ACRs instead of enhancers. Enhancer means a distal element that is able to interact with the promoter; thus, without such data, the word enhancer should not be used.

***Response:*** *Thank you for your suggestions. We agree with your opinion that the description of putative enhancer in this article is not so rigorous. Therefore, we modified the description of the relevant parts and unified the description of “putative enhancer” into “putative regulatory elements” or “distal ACRs (dACRs)”.*

Similarly, in Fig. 4f and g, the author examined the GWAS signals of certain traits within 1kb of identified regulatory ACRs. Although the data do support those regulatory elements are generally meaningful, there is no data to support so-called enhancers are correct, a comparison of enhancers versus non-enhancers but with similar chromatin accessibility is missing here. In other words, regulatory ACRs proximal or distal to the gene are linked with traits, unsurprisingly, but no claim can be made about whether they are enhancers.

***Response:*** *Thank you for your comments. We agree with your opinion, and we modified the description of the relevant parts and unified the description of “putative enhancer” into “putative regulatory elements” or “distal ACRs (dACRs)”.*

The authors performed a LUC assay to validate the enhancers in Fig 4i. I found this assay problematic. The assay just simply puts an ACR immediately before a mini-promoter. The spatial information is lost in this case. Is there any negative control that a certain ACR can not activate the transcription of the reporter? I doubt that.

***Response:*** *Thank you for your comments. Regarding the issue of spatial information, adding spatial settings will indeed explain remote control more perfectly. However, in terms of experimental operation, it is almost impossible for us to add fragments between ACR and mini-promoter to verify the long-range interaction. And the vector construction method we adopted is similar to the protocol in previously published articles (Lin Y, et al: Rapid validation of transcriptional enhancers using agrobacterium-mediated transient assay. Plant Methods 2019, 15.; Xie YL, et al: Enhancer transcription detected in the nascent transcriptomic landscape of bread wheat. Genome Biol 2022, 23.).*

*As you suggested, we picked up two other regions with high accessibility but no variations during vernalization (IGV screenshots below), and checked their regulatory activities. It’s found that results showed that they did not respond to vernalization (the bar chart below).*

*Figure for response (Fig. S6c of the revised version). IGV screenshots of two vernalization-unresponsive ACRs. CK1 and CK2 were designed based on the ACRs who were not respond to vernalization.*

*Figure for response (Fig. S6d of the revised version). Transcriptional regulatory activities of CK1 and CK2 through luciferase (LUC) activity assays in wheat protoplasts.*

9. The author argued that the SPL7/15 regulates vernalization, yet the flowering time phenotype of the mutant is mainly before vernalization. This seems to argue that these genes mainly do not function during vernalization. Nevertheless, more discussion is needed at least.

***Response:*** *Thank you for your comments. Our study revealed that SPL7 and SPL15 can bind to the regulatory regions of VRN1 and VRN3, leading to an increase in their expression during vernalization. However, despite this increase in expression, the late flowering phenotype of spl7-aabbdd spl15-aabbdd hexa-mutants was still suppressed by elongating cold vernalization treatment. We hypothesize that there may be an SPL-independent pathway responsible for the up-regulation of VRN1 and VRN3. This pathway could lead to the accumulation of high levels of VRN1, thereby rescuing the late flowering phenotype observed in spl7-aabbdd spl15-aabbdd hexa-mutants. In essence, VRN1 may serve as a critical node in balancing the two signaling pathways, including age-dependent pathways, thus influencing the overall flowering response. To better discuss these findings, we added the following descriptions in discussion (PAGE 19, LINE 458-462): “**We verified that while TaSPL7/15 is induced by vernalization, it participates in the wheat vernalization process by regulating VRN1 and VRN3. Although the deficiency of SPL7 and SPL15 delays the expression rise of VRN1 and VRN3, the late flowering phenotype of spl7a/b/d spl15a/b/d hexa-mutants can be suppressed by extending the period of cold* *vernalization. This indicates that there is an SPL7/15-independent pathway that can activate VRN1 and VRN3 expression.”*

Minor concerns:

10. Genome browser tracks of individual repeats should be displayed to demonstrate the reproducibility of the data better.

***Response:*** *Thank you for your suggestion. We have shown two duplicates in all IGV screenshots, including Fig. 1b, Fig. 2h, Fig. 4c, Fig. 5f, Fig. 6c, Fig. S2a, Fig. S5c, Fig. S 6a, and Fig. S8c.*

11. Line 54，"cold signal triggers SAM transition to reproductive SAM…," a reference is needed here.

***Response:*** *Thank you for your suggestion. We have added a reference.*

12. Line 57, the nature of VRN2 protein should be briefly introduced.

*Response: Thank you for your suggestion. We have added a description of VRN2 protein “VRN2, codes a zinc finger protein, represses flowering and its disruption leads to unvernalized flowering (Yan L, et al: The wheat VRN2 gene is a flowering repressor down-regulated by vernalization. Science 2004, 303:1640-1644.; Distelfeld A, et al: Genetic and molecular characterization of the VRN2 loci in tetraploid wheat. Plant Physiol 2009, 149:245-257.; Kippes N, et al: Development and characterization of a spring hexaploid wheat line with no functional VRN2 genes. Theor Appl Genet 2016, 129:1417-1428.)”.*

13. Line 70, there should be no H3K9 at FLC; the results from some early-year publications might not be accurate. please remove such a claim.

***Response:*** *Thank you for your suggestions. We have removed this statement in the revised version.*

14. Related to Fig1b and Fig. S2, what's the difference among VRN1A/B/D in terms of histone modifications before and after vernalization? This should be described and displayed at least as supplementary data.

***Response:*** *Thank you for your suggestion. We have added figures of VRN1 chromatin accessibility and histone modifications on the B and D sub-genomes in Fig. S2a, and discussed the changes of VRN1 on the A, B and D genomes in the main text “Stronger chromatin accessibility signals were found in the promoter of VRN1, and histone modifications H3K27ac, H3K4me3, and H3K36me3 were also increased in A and B sub-genome, H3K27ac and H3K4me3 were increased in D sub-genome, while H3K27me3 was decreased after vernalization (Fig. 1b and Fig. S2a)”.*

15. Line 210-212, citations are needed here.

***Response:*** *Thank you for your suggestion. We have added a citation.*

16. Line 217 to Line 219, examples of different modes of subgenome-specific vernalization responses should be displayed, at least in the supplementary data.

***Response:*** *Thank you for your suggestion. In the revised Fig. S5d we have shown two examples and added images to the text description (PAGE 11, LINE 243-245). “Among the expressed genes (TPM >=1 in at least one tissue), 55% showed balanced responses among the three subgenomes, while 16% showed unbalanced responses (Fig. 3c-d and Fig. S5d)”.*

17. Line 283-286, the connection with the previous paragraph is quite odd. Please consider re-writing this part.

***Response:*** *Thank you for your suggestion. We have re-described the contents here “In addition, we characterized the states of vernalization-responsive nK4-dACRs before and after vernalization. Among all these ACRs with state changes, the most common ones are transitioned from activated to unmodified, repressed to unmodified, unmodified to activated and unmodified to repressed, then mixed to repressed, and mixed to unmodified (Fig. 4j), suggesting a consistent pattern with that of genes”.*

18. Figure 6f and g, what is P5? Figure 6g: where are the results of P2? Figure 4i, what are the P1 to P5? The information or results are missing and should be added.

***Response:*** *Thank you for your comments.*

*For P1 to P5 in Figure 4i, we have added their information in figure legend “P1~P4* *are the ACRs marked in Fig. 4c, and P5 is the ACR marked in Fig. S6a”.*

*As for figure 6f and g, we also added the information of these fragments “P1~P4 are the ACRs marked in Fig. 6c, and P5 is the ACR marked in Fig. S8d”.*

*The results of EMSA in Fig. 6e showed that SPL did not bind P2, so no dual luciferase Reporter assay experiments with P2 were showed before. And the following picture was the result of P2.*

*Figure for response (Fig. S8f of the revised version). Validation of TaSPL7/15 specific* *binding. P2 was marked in Fig. 4c.*

19. Please carefully update the figure legends. Currently, it is difficult to read as much information was omitted. Many abbreviations were not properly defined.

***Response:*** *Thank you for your comments. We checked the figure legend of all the pictures and made necessary modifications. For abbreviations, we also double checked and added the full-text.*

**Reviewer 1 and 3**

Overall, the authors have sufficiently addressed the issues I raised by provided new results and revisions to the text. However, some points still need to be corrected to improve the manuscript.

As pointed out in the first round of review, when evaluating flowering time using leaf number, it is most informative from a developmental perspective to present the number of leaves on the parent culm rather than the total leaf number. The reason: if the mutants have more or fewer secondary branches than WT, they will have more or fewer total leaves as a result of this developmental difference in branching pattern rather than a developmental difference in when flowering is initiated, and thus total leaf number could misrepresent flowering time. However, I think it is acceptable for the authors to use the total leaf number data if a clear statement of what was done is in the legend to Fig. S9. Furthermore, in the manuscript that was provided for the second round of review there is not a legend for any of Fig. S9!

The authors must provide a complete legend for Fig. S9 and in that legend it is important to make sure two things are addressed:

1. Fig. S9b legend: Make it clear that leaf number represents the total number of leaves on all branches counted at [DESCRIBE THE DEVELOPMENTAL STAGE OF FLOWERING WHEN LEAF COUNTS WERE DONE].
2. Fig. S9c: It appears that the WT has flowered which indicates that the plants were subjected to a vernalization treatment. Provide details on the conditions under which the plants in the image were grown including whether the plants were vernalized by cold exposure prior to growing in warm conditions.

**Reviewer 2**

The authors have addressed my concerns. I have no further questions.

**Authors Response**

**Point-by-point responses to the reviewers’ comments:**

Reviewer #1&3:
Overall, the authors have sufficiently addressed the issues I raised by provided new results and revisions to the text. However, some points still need to be corrected to improve the manuscript.
*Response: We appreciate your professional comments and have made the changes according to your suggestions.*
As pointed out in the first round of review, when evaluating flowering time using leaf number, it is most informative from a developmental perspective to present the number of leaves on the parent culm rather than the total leaf number. The reason: if the mutants have more or fewer secondary branches than WT, they will have more or fewer total leaves as a result of this developmental difference in branching pattern rather than a developmental difference in when flowering is initiated, and thus total leaf number could misrepresent flowering time. However, I think it is acceptable for the authors to use the total leaf number data if a clear statement of what was done is in the legend to Fig. S9. Furthermore, in the manuscript that was provided for the second round of review there is not a legend for any of Fig. S9! The authors must provide a complete legend for Fig. S9 and in that legend it is important to make sure two things are addressed:
1. Fig. S9b legend: Make it clear that leaf number represents the total number of leaves on all branches counted at [DESCRIBE THE DEVELOPMENTAL STAGE OF FLOWERING WHEN LEAF COUNTS WERE DONE].
*Response: We appreciate your comments and have added the legend to Fig. S9 with detailed information about plant conditions and the time documenting the phenotypes.
“Fig. S9. Heading time and leaf counts of ZM7698 and spl7-aabbdd spl15-aabbdd hexa-mutant. a. Heading times of ZM7698, spl7-aabb spl15-aabbdd, and spl7-aabbdd spl15-aabbdd mutants. All plants were treated with 2, 3, and 4 weeks of vernalization (4°C, 16 h light/8 h dark) after growing in the greenhouse (day for 16h at 22°C, night for 8h at 19°C) for 2 weeks. After vernalization, the plants continued to grow in the greenhouse (day for 16h at 22°C, night for 8h at 19°C), and the heading time of the earliest tiller of each plant was documented. b. Leaf counts of ZM7698 and spl7-aabbdd spl15-aabbdd hexa-mutant from germination to heading. All plants were treated with 3 weeks of vernalization (4°C, 16 h light/8 h dark) after growing in the greenhouse (day for 16h at 22°C, night for 8h at 19°C) for 2 weeks. After vernalization, all plants continued to grow in the greenhouse (day for 16h at 22°C, night for 8h at 19°C). The total leaf counts of all branches were recorded every two days until the heading of ZM7698. c. Phenotypes of single tillers. The plants were grown under the same conditions as described in b. The photos were taken when ZM7698 was about to head (70 days after germination).”.*

2. Fig. S9c: It appears that the WT has flowered which indicates that the plants were subjected to a vernalization treatment. Provide details on the conditions under which the plants in the image were grown including whether the plants were vernalized by cold exposure prior to growing in warm conditions.
*Response: We appreciate your comments. We have added the legend with detailed growing conditions to Fig. S9c according to your suggestions.*

Reviewer #2:
The authors have addressed my concerns. I have no further questions.
*Response: We appreciate your professional comments and your efforts to help improve our manuscript.*
